# Modeling the vacuolar storage of malate shed lights on pre- and post-harvest fruit acidity

**DOI:** 10.1186/s12870-014-0310-7

**Published:** 2014-11-18

**Authors:** Audrey Etienne, Michel Génard, Philippe Lobit, Christophe Bugaud

**Affiliations:** Centre de Coopération International en Recherche Agronomique pour le Développement (CIRAD), UMR QUALISUD, Campus Agro-Environnemental Caraïbe, BP 214, 97 285 Lamentin, Cedex 2 France; INRA, UR 1115 Plantes et Systèmes de Cultures Horticoles, F-84914 Avignon, France; Instituo de investigaciones Agropecuarias y Forestales, Universidad Michoacana de San Nicolás de Hidalgo, Tarímbaro, Michoacán CP 58880 Mexico; CIRAD, UMR QUALISUD, TA B-95 /16, 73 rue Jean-François Breton, 34398 Montpellier, Cedex 5 France

**Keywords:** Banana, Cultivar, Fruit acidity, Malic acid, Model, *Musa*, Organic acid, Potassium, Pre- and post-harvest, Vacuolar storage

## Abstract

**Background:**

Malate is one of the most important organic acids in many fruits and its concentration plays a critical role in organoleptic properties. Several studies suggest that malate accumulation in fruit cells is controlled at the level of vacuolar storage. However, the regulation of vacuolar malate storage throughout fruit development, and the origins of the phenotypic variability of the malate concentration within fruit species remain to be clarified. In the present study, we adapted the mechanistic model of vacuolar storage proposed by Lobit *et al*. in order to study the accumulation of malate in pre and postharvest fruits. The main adaptation concerned the variation of the free energy of ATP hydrolysis during fruit development. Banana fruit was taken as a reference because it has the particularity of having separate growth and post-harvest ripening stages, during which malate concentration undergoes substantial changes. Moreover, the concentration of malate in banana pulp varies greatly among cultivars which make possible to use the model as a tool to analyze the genotypic variability. The model was calibrated and validated using data sets from three cultivars with contrasting malate accumulation, grown under different fruit loads and potassium supplies, and harvested at different stages.

**Results:**

The model predicted the pre and post-harvest dynamics of malate concentration with fairly good accuracy for the three cultivars (mean RRMSE = 0.25-0.42). The sensitivity of the model to parameters and input variables was analyzed. According to the model, vacuolar composition, in particular potassium and organic acid concentrations, had an important effect on malate accumulation. The model suggested that rising temperatures depressed malate accumulation. The model also helped distinguish differences in malate concentration among the three cultivars and between the pre and post-harvest stages by highlighting the probable importance of proton pump activity and particularly of the free energy of ATP hydrolysis and vacuolar pH.

**Conclusions:**

This model appears to be an interesting tool to study malate accumulation in pre and postharvest fruits and to get insights into the ecophysiological determinants of fruit acidity, and thus may be useful for fruit quality improvement.

**Electronic supplementary material:**

The online version of this article (doi:10.1186/s12870-014-0310-7) contains supplementary material, which is available to authorized users.

## Background

Malate is one of the most important organic acids in many fruits [1], and its concentration in the pulp plays a critical role in organoleptic properties [2-4]. The malate concentration varies considerably among cultivars of many fruit species including peach [5], apples [6,7] and loquat [8]. The malate concentration undergoes great changes during fruit growth [9,10] and also during postharvest ripening [11,12]. Understanding the mechanisms that control malate accumulation is thus of primary importance for fruit quality improvement.

The accumulation of malate in fruit cells is a complex phenomenon because it involves several metabolic pathways and transport mechanisms across different compartments, mainly cytosol, mitochondria, and vacuole. Concerning malate, we showed in a previous paper [13] that the thermodynamic conditions of its transport into the vacuole may limit its accumulation. Therefore, one can hypothesize that malate accumulation in fruit cells is mainly controlled at the level of vacuolar storage, and that metabolism responds appropriately to regulate the cytosolic concentration of malate since it plays a fundamental role in the regulation of cytosolic pH [14]. However, the regulation of vacuolar malate storage throughout fruit development, and the origins of the phenotypic variability of the malate concentration within fruit species remain to be clarified. Given the complexity of the processes, ecophysiological process-based simulation models (PBSMs) could advance our understanding of the mechanisms underlying malate accumulation in pre and postharvest fruits. PBSMs could also help to elucidate the differences in malate accumulation among cultivars, as was the case for sugar accumulation in peach [15], and grape berry [16].

Despite the importance of pulp malate concentration for fruit quality, attempts to mechanistically model it are rare. To our knowledge, the only PBSM was proposed by Lobit *et al*. [17] to simulate malate concentration in peach. This model is based on a simplified representation of the functioning of the tonoplast to simulate vacuolar malate storage and thus appears to be a good framework to study malate accumulation in fleshy fruit.

In the present study, we adapted Lobit’s model in order to study the accumulation of malate in pre and postharvest fruit using a mechanistic model-based analysis. The main adaptation concerned the variation of the free energy of ATP hydrolysis during fruit development. Banana fruit was taken as a reference because it has the particularity of having separate growth and post-harvest ripening stages, during which malate concentration undergoes substantial changes [18]. Moreover, the concentration of malate in banana pulp varies greatly among cultivars which make possible to use the model as a tool to analyze the genotypic variability [11,19]. The physiological age of the fruit at harvest is known to affect the concentration of malate in the pulp of banana during post-harvest ripening [20]. Fruit pruning and potassium fertilization, two cultural practices commonly used by the banana growers, can also impact the concentration of malate in fleshy fruits (for review see [13]). Consequently, we chose to calibrate and validate the model on three cultivars with contrasting malate accumulation, grown under different fruit loads and potassium supplies, and harvested at different stages. To study how these factors could affect malate accumulation, we analyzed the sensitivity of the model to parameters and input variables. The model enabled us to: improve our understanding of malate accumulation during growth and post-harvest ripening of fruit; propose a possible explanation for differences in malate accumulation among cultivars; study the possible effects of fruit growth conditions on malate accumulation. Finally, this model appears to be an interesting tool to study malate accumulation in pre and postharvest fruits and to get insights into the ecophysiological determinants of fruit acidity, and thus may be useful for fruit quality improvement.

## Methods

### Model description

**Figure 1:**
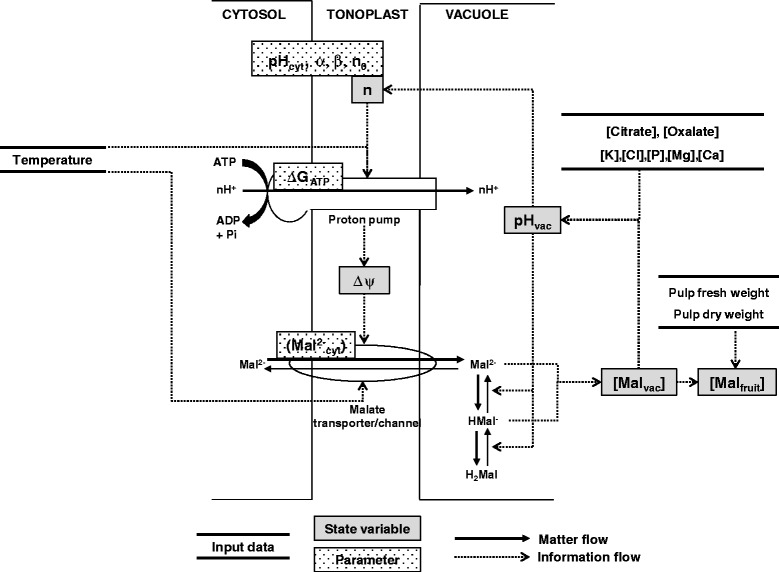
**Schematic representation of the model of vacuolar malate storage proposed by Lobit** ***et al*** **. (2006) [** 17 **].** State variables: [Mal_fruit_] = concentration of malate in the pulp; [Mal_vac_] = concentration of malate in the vacuole; pH_vac_ = vacuolar pH; ΔΨ = electric potential gradient across the tonoplast; n = coupling ratio of the proton pump ATPase. Model parameters: pH_cyt_ = cytosolic pH; ΔG_ATP_ = free energy of ATP hydrolysis; α, β, and n_0_ = fitted parameters of the coupling ratio equation (Eq. 5); (Mal^2−^_cyt_) = cytosolic activity of the di-anion malate.

The model of malate accumulation proposed by Lobit *et al*. [17] assumes that the accumulation of malate in fleshy fruits is mainly determined by the conditions of its storage in the vacuole of pulp cells. The model provides a simplified representation of the functioning of the tonoplast (Figure 1).

The transport of malate across the tonoplast is passive and occurs by facilitated diffusion of the di-anion form through specific ion channels [21-23] and transporters [24,25]. It follows the electrochemical potential gradient of the di-anion across the tonoplast, defined as follows:

1 $$ {{\Delta \mathrm{G}}_{\mathrm{Mal}}}^{2\hbox{-} }=-2\mathrm{F}\Delta \Psi +\mathrm{RTln}\left(\left({{\mathrm{Mal}}^{2\hbox{-}}}_{\mathrm{vac}}\right)/\left({{\mathrm{Mal}}^{2\hbox{-}}}_{\mathrm{cyt}}\right)\right) $$

where (Mal^2−^_cyt_) and (Mal^2−^_vac_) are the activities of the di-anion malate in the cytosol and in the vacuole respectively (mol L^−1^), ΔΨ is the electric potential gradient across the tonoplast (ψ_vac_-ψ_cyt_; V), T is temperature (K), R is the gas constant (8.3144621 J mol^−1^ K^−1^), and F is Faraday’s constant (9.65∗10^4^ C mol^−1^).

This implies that the accumulation of malate in the vacuole is controlled mainly by the ratio of the di-anion malate activity across the tonoplast and the ΔΨ.

The activity of the di-anion is the product of its activity coefficient a_Mal_^2−^ (dimensionless) and of its concentration [Mal^2−^] (mol L^−1^):

2 $$ \left({\mathrm{Mal}}^{2-}\right)={{\mathrm{a}}_{\mathrm{Mal}}}^{2-}\ast \left[{\mathrm{Mal}}^{2-}\right] $$

In the cytosol, the concentration of the di-anion malate is unlikely to vary much because it plays a fundamental role in the regulation of cytosolic pH [14]. In addition, its activity coefficient, which depends only on the ionic strength of the cytosol, is also unlikely to vary much [17]. Therefore, in the model, (Mal^2−^_cyt_) is considered as a constant.

In the vacuole, the activity coefficient of the di-anion malate (a_Mal_^2−^_vac_) is related to the concentration of all ionic species [18], while its concentration is proportional to the total malate concentration and is controlled by the dissociation equation, since malate is a weak acid:

3 $$ \left[{{\mathrm{Mal}}^{2-}}_{\mathrm{vac}}\right]=\left[{\mathrm{Mal}}_{\mathrm{vac}}\right]\ast \left(\left(\mathrm{K}{\prime}_1\mathrm{K}{\prime}_2\right)/\left({\mathrm{h}}^2+\mathrm{h}\mathrm{K}{\prime}_1+\mathrm{K}{\prime}_1\mathrm{K}{\prime}_2\right)\right) $$

where [Mal_vac_] is the total concentration of malate in the vacuole (mol L^−1^), h = 10^-pHvac^, and K′_1_ and K′_2_ are the apparent acidity constants of malate (mol L^−1^).

In plant cells, ΔΨ is mainly generated by the tonoplastic proton pumps, which catalyze the active transport of protons into the vacuole. Two types of pumps are present on the tonoplast of fruit cells: the ATPase [26] and the PPiase [27], which respectively hydrolyze ATP and PPi as a source of energy. Both are known to be active in most fruits [24,28,29], but for the sake of simplicity, only ATPase was taken into account in the model. Proton pumping can occur only if the variation in free energy of the chemiosmotic reaction ΔG_ATPase_ defined below is negative:

4 $$ \Delta {\mathrm{G}}_{\mathrm{ATP}\mathrm{ase}}=\Delta {\mathrm{G}}_{\mathrm{ATP}}+\mathrm{nF}\Delta \Psi -\mathrm{nRTln}(10)\ast \left({\mathrm{pH}}_{\mathrm{vac}}-{\mathrm{pH}}_{\mathrm{cyt}}\right) $$

where ΔG_ATP_ is the free energy of ATP hydrolysis (J mol^−1^), n is the coupling ratio i.e. the number of protons pumped by hydrolyzed ATP, pH_vac_ and pH_cyt_ are vacuolar and cytosolic pH respectively.

The pH gradient across the tonoplast plays a role in this equation, both directly, and because it affects the coupling ratio n. Lobit *et al*. [17] fitted the following equation to the data of Davies *et al*. [30] to calculate the coupling ratio:

5 $$ \mathrm{n}={\mathrm{n}}_0+\upalpha \left({\mathrm{pH}}_{\mathrm{vac}}-7\right)+\upbeta {10}^{\left(\mathrm{pHcyt}-7\right)} $$

where n_0_, α, and β are fitted parameters.

The approach used in this model is to represent changes in vacuolar composition as a succession of stationary states during which malate concentration, pH_vac_, and ΔΨ can be considered to be constant. The assumption is that the transport of the di-anion malate and protons operate in conditions close to their respective thermodynamic equilibrium.

Assuming that the di-anion malate is at thermodynamic equilibrium across the tonoplast implies that ΔG_Mal_^2−^ = 0. So rewriting and combining equations 1, 2 and 3 gives:

6 $$ \left[{\mathrm{Mal}}_{\mathrm{vac}}\right]=\left(1/{\mathrm{a}}_{\mathrm{Mal}}{{}^{2-}}_{\mathrm{vac}}\right)\ast \left(\left({\mathrm{h}}^2+\mathrm{h}\mathrm{K}{\prime}_1+\mathrm{K}{\prime}_1\mathrm{K}{\prime}_2\right)/\left(\mathrm{K}{\prime}_1\mathrm{K}{\prime}_2\right)\right)\ast \left({{\mathrm{Mal}}^{2-}}_{\mathrm{cyt}}\right)\ast \exp \left(2\mathrm{F}\Delta \Psi /\mathrm{R}\mathrm{T}\right) $$

Assuming that proton transport occurs at thermodynamic equilibrium implies that ΔG_ATPase_ = 0. So, rewriting and combining equations 4 and 5 gives:

7 $$ \Delta \Psi =\left(-\varDelta {\mathrm{G}}_{\mathrm{ATP}}/\left(\left({\mathrm{n}}_0+\upalpha \left({\mathrm{pH}}_{\mathrm{vac}}-7\right)+\upbeta {10}^{\left(\mathrm{pHcyt}-7\right)}\right)\mathrm{F}\right)\right)+\left(\mathrm{R}\mathrm{T}/\mathrm{F}\right)\ast \ln (10)\ast \left({\mathrm{pH}}_{\mathrm{vac}}-{\mathrm{pH}}_{\mathrm{cyt}}\right) $$

The acid/base composition of the vacuole determines a_Mal_^2−^_vac_, K′_1_, K′_2_, and pH_vac_. These variables are calculated using a model of pH prediction that was described and validated on banana fruit in a previous paper [18]. As input variables, the model requires the concentrations of the three main organic acids present in banana pulp, citrate, malate, and oxalate (oxalate being present in large amounts at the green stage [18]), and of the main soluble mineral elements, namely potassium, magnesium, chloride, calcium, and phosphorus.

Solving the malate model means solving a system of equations with two unknowns, [Mal_vac_] and pH_vac_, and six parameters, pH_cyt_, (Mal^2−^_cyt_), ΔG_ATP_, n_0_, α, and β. Once the concentration of malate in the vacuole is determined, the concentration of malate in the pulp can be calculated by assuming that the volume of water in the vacuole is equal to the water mass of the pulp:

8 $$ \left[{\mathrm{Mal}}_{\mathrm{fruit}}\right]=\left[{\mathrm{Mal}}_{\mathrm{vac}}\right]\ast \left(\left(\mathrm{F}\mathrm{W}-\mathrm{D}\mathrm{W}\right)/\mathrm{F}\mathrm{W}\right)\ast 1000 $$

where [Mal_fruit_] is the concentration of malate in the pulp (mmol Kg FW^−1^), FW and DW are the pulp fresh weight and pulp dry weight respectively (g).

### Changes in ΔG_ATP_ during banana development

According to the sensitivity analysis of the model performed by Lobit *et al*. [17] on peach, malate accumulation is strongly dependent on ΔG_ATP_. According to the literature, ΔG_ATP_ can vary considerably depending on cytosolic conditions [31,32], so that one may expect ΔG_ATP_ to vary during banana development. The possible variation of ΔG_ATP_ required (according to the model) to sustain malate accumulation during banana growth and postharvest ripening was assessed by reorganizing and combining equations 6 and 7, and by assuming that pH_cyt_ = 7 (common notion of a neutral cytosol), (Mal^2−^_cyt_) =0.001 mol L^−1^ (reasonable value according to Lobit *et al*. [17]), a_Mal_^2−^_vac_ =0.3 (average value found by the banana pH model [18]), and parameters n_0_ = 4, α = 0.3, and β = −0.12 (to calculate n with equation 5) [17].

9 $$ \Delta {\mathrm{G}}_{\mathrm{ATP}}=\mathrm{nRTln}(10)\ast \left({\mathrm{pH}}_{\mathrm{vac}}-{\mathrm{pH}}_{\mathrm{cyt}}\right)-\left(\mathrm{n}\mathrm{R}\mathrm{T}/2\right)\ast \ln \left(\left(\mathrm{K}{\prime}_1\mathrm{K}{\prime}_2\left[{\mathrm{Mal}}_{\mathrm{vac}}\right]{\mathrm{a}}_{\mathrm{Mal}}{{}^{2-}}_{\mathrm{vac}}\right)/\left(\left({\mathrm{h}}^2+\mathrm{h}\mathrm{K}{\prime}_1+\mathrm{K}{\prime}_1\mathrm{K}{\prime}_2\right)\left({{\mathrm{Mal}}^{2-}}_{\mathrm{cyt}}\right)\right)\right) $$

**Figure 2:**
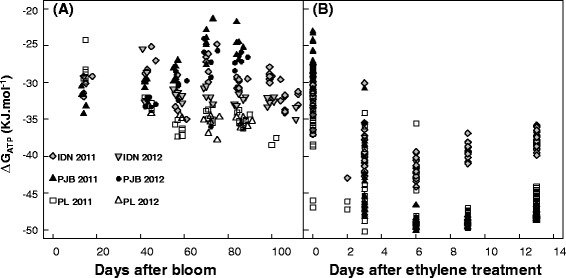
**Variations in ΔG**_**ATP**_ **during fruit development for cultivars IDN, PJB, and PL.** ΔG_ATP_ were plotted as a function of **(A)** days after bloom during fruit growth, and **(B)** days after ethylene treatment during post-harvest ripening. These values were calculated with equation 9 using the data for the three cultivars for 2011 and 2012.

Changes in ΔG_ATP_ over time, calculated with equation 9 and using 12 datasets including three cultivars, two developmental stages (pre- and post-harvest stage), and 2 years, were plotted. During fruit growth, ΔG_ATP_ varied little (Figure 2A) whereas during post-harvest ripening, there was a negative relationship between ΔG_ATP_ and the number of days after ethylene treatment in all three cultivars (Figure 2B). Thus, we considered ΔG_ATP_ as a constant during fruit growth and simulated the observed relationship with days after ethylene treatment during ripening by the following function:

10 $$ \Delta {\mathrm{G}}_{\mathrm{ATP}}={\mathrm{G}}_1\ast \mathrm{D}\mathrm{A}{\mathrm{E}}^2+{\mathrm{G}}_2\ast \mathrm{D}\mathrm{A}\mathrm{E}+{\mathrm{G}}_3 $$

where DAE is the day after ethylene treatment, and G_1_ (J mol^−1^ day^−2^), G_2_ (J mol^−1^ day^−1^), and G_3_ (J mol^−1^) are fitted parameters.

### Model inputs

The input variables required were temperature (T; K), pulp fresh weight (FW; g), pulp dry weight (DW; g), pulp potassium content (K; mol L^−1^), pulp magnesium content (Mg; mol L^−1^), pulp phosphorus content (P; mol L^−1^), pulp calcium content (Ca; mol L^−1^), pulp chloride content (Cl; mol L^−1^), pulp citrate content (mol L^−1^), and pulp oxalate content (mol L^−1^).

### Plant materials and experimental conditions

All experiments were conducted at the *Pôle de Recherche Agroenvironnementale de la Martinique* (PRAM, Martinique, French West Indies; latitude 14°37 N, longitude 60°58 W, altitude 16 m) using three cultivars of dessert banana (Musa spp.) diploids AA, differing in predominant organic acid at the eating stage: Indonesia 110 (IDN), Pisang Jari Buaya (PJB), and Pisang Lilin (PL). The plant material is deposited at the *in vitro* collection of Bioversity International (Bioversity International Transit Center c/o KU Leuven, Division of Crop Biotechnics, Laboratory of Tropical Crop Improvement Willem de Croylaan; 42 box 2455, BE3001 Heverlee, Belgium) under the internal codes ITC0712, ITC0690, ITC1121 respectively. Bioversity International Transit Center collection is an FAO ‘in trust’ collection for which Bioversity has the commitment to ensure the long term storage of holdings and provide unrestricted access by the Musa community. The collection is part of the multilateral system of the International Treaty on Plant Genetic Resources for Food and Agriculture. Experiments were conducted during the 2011 and 2012 growing seasons on continental alluvial soil. In both growing seasons, irrigation was adjusted to the amount of rainfall to supply at least 5 mm of water per day, and non-systemic fungicide was applied to control foliar diseases. During the first period of bunch growth (March–November 2011) the mean daily temperature was 27 ± 1.2°C. During the second period of bunch growth (February–August 2012) the mean daily temperature was 26 ± 0.9°C.

#### 2011 experiment: effect of fruit load on banana pulp acidity

For each cultivar, 36 plants were randomly chosen and tagged at inflorescence emergence. Two contrasted fruit loads were used: 18 plants of each cultivar were used as the control treatment i.e. high fruit load, and 18 other plants were highly pruned i.e. low fruit load. In the control treatment, the number of leaves and hands left on the plants were calculated in order to have the same leaf area: fruit ratio among cultivars (approximately equal to 0.5 cm^2^ leaf. g fruit^−1^). Thus, 15 days after inflorescence emergence, 8, 6, and 5 leaves were left on the plant for cultivars IDN, PL, and PJB respectively, and the top 10, 5 and 7 hands were left on the bunch for cultivars IDN, PL, and PJB respectively. To ensure the situation was the same among the three cultivars, fruit pruning in low fruit load treatment was calculated to increase the leaf area: fruit ratio by approximately 2.5. Consequently, 15 days after inflorescence emergence, the top 4, 2, and 3 hands were left on the bunch for cultivars IDN, PL, and PJB respectively. Banana plants received 12 g of nitrogen, 1.7 g of phosphorus, and 23 g of potassium at 4-week intervals during fruit growth.

#### 2012 experiment: effect of potassium fertilization on banana pulp acidity

Two plots containing 50 banana plants of each cultivar were planted. Two contrasted levels of potassium fertilization were started six months before the beginning of fruit sampling. For each cultivar, one plot received 124 g of potassium per plant (high potassium fertilization) at 4-week intervals, while the other received no potassium at all. All the banana plants received 12 g of nitrogen and 10 g of phosphorus at 4-week intervals. Twenty-four plants of each cultivar were randomly chosen in each plot and tagged at inflorescence emergence. At 15 days after inflorescence emergence, 9, 7, and 9 leaves were left on cultivars IDN, PL, and PJB respectively, which corresponded to the average leaf number in 2012, and the top 10, 5, and 7 hands were left on the bunch of cultivars IDN, PL, and PJB respectively, which corresponded to a high fruit load.

#### Fruit growth monitoring

In the two growing seasons, six bunches were selected for each cultivar∗treatment combination. One fruit located in the internal row of the second proximal hand was collected for analyses every 15 days. Natural ripening on standing plants, i.e. when the first yellow finger appears, determined the end of sampling.

#### Monitoring of post-harvest ripening

In the 2011 experiment, two harvest stages were studied. The stages were calculated so that each cultivar was at 70% and 90% of the average flowering-to-yellowing time (FYT) of the bunch on the tree. At each harvest stage, six bunches per cultivar and per treatment were harvested. In the 2012 experiment, only one harvest stage was studied. For each cultivar, this stage was calculated to be 75% of the average FYT of the bunch on the tree. Six bunches per cultivar and per treatment were harvested. After the bunches were harvested, the second proximal banana hand per bunch was rinsed and dipped in fungicide (bitertanol, 200 mg L^−1^) for 1 min. The fruits were placed in a plastic bag with 20 μm respiration holes and stored in boxes for 6 days at 18°C. The fruits were then stored in a room at 18°C and underwent ethylene treatment (1 mL L^−1^ for 24 h) to trigger the ripening process. After 24 h, the room was ventilated. Bananas were maintained at 18°C during 13 days. One banana fruit was sampled before ethylene treatment, and at day 3, 6, 9 and 13 after ethylene treatment.

### Biochemical measurements

The fresh and dry pulp of each sampled fruit was weighed. The dried pulp was then ground to obtain a dry powder for biochemical measurements. Citric acid and malic acid concentrations were determined according to Etienne *et al*. [18] using an enzymatic method and a microplate reader. The soluble oxalic acid concentration was determined using the LIBIOS Oxalic acid assay kit. Pulp soluble K, Mg, and Ca concentrations were determined by mass spectrometry, and soluble P was measured by colorimetry [33]. The Cl concentration in the pulp was determined by potentiometry using the automatic titrator TitroLine alpha [34].

### Model solving and parameterization

The model was computed using R software (R Development Core Team, http://www.r-project.org) (Additional files 1, 2, 3, 4 and 5). For each sampling date, the system was solved to calculate the concentration of malate in the pulp, using the “nleqslv” function of the R software, which solves a system of non-linear equations using a Broyden method (http://cran.r-project.org/web/packages/nleqslv/index.html). (Mal^2−^_cyt_) was set at 0.001 mol L^−^1 which is within the range mentioned by Lobit *et al*. [17]. pH_cyt_ was set at 7 according to the common notion of a neutral cytosol. For parameters n_0_, α, and β, which define the stoechiometry of the pump ATPase, Lobit *et al*. [17] estimated values very close to those found by fitting equation 5 to the data of Davies *et al*. [30] and Kettner *et al*. [35]. This suggests that these parameters correspond to a structural characteristic of ATPase and are unlikely to vary much, so we chose to set them to the values found by Lobit *et al*. [17] (Table 1).

**Table 1:** **Values of model parameters**

**Parameter**	**Value**			**Unit**	**Description**	**Origin**
	**IDN**	**PJB**	**PL**			
pH_cyt_	7			Unit pH	Cytosolic pH	Literature
(Mal^2−^_cyt_)	0.001			mol L^−1^	Cytosolic activity of the di-anion malate	Literature
n_0_	4			dimensionless	Parameters to calculate the coupling ratio of the proton pump	Literature
α	0.3			dimensionless		Literature
β	−0.12			dimensionless		Literature
ΔG_ATP_	−36.9∗10^3^	−39.1∗10^3^	−47.4∗10^3^	J mol^−1^	Free energy of ATP hydrolysis during banana growth	Estimated
G_1_	75	69	110	J mol^−1^ day^−2^	Parameters to calculate ΔG_ATP_ as a function of the number of days after ethylene treatment during banana post-harvest ripening	Estimated
G_2_	−1176	−1108	−1959	J mol day^−1^		Estimated
G_3_	−45.2∗10^3^	−48.9∗10^3^	−46.3∗10^3^	J mol^−1^		Estimated

### Model calibration

Parameter ΔG_ATP_ was estimated by fitting the model to observed values of the pre-harvest 2011 dataset separated by cultivar (24 < *n* < 36) (Additional file 6). Parameters G_1_, G_2_, and G_3_ were estimated by fitting the model to ΔG_ATP_ values calculated according to equation 9 from the 2011 post-harvest dataset separated by cultivar (54 < *n* < 60). The harvest stage was not taken into account since there were no differences in the variations in ΔG_ATP_ calculated with equation 9 between fruits harvested at 70% and 90% of FYT (data not shown). Parameters were estimated using the “hydroPSO” function of R software [36]. The hydroPSO function uses the computational method of particle swarm optimization (PSO) that optimizes a problem by iteratively trying to improve a candidate solution with regard to a given measure of quality. Parameters were estimated by minimizing the following criterion:

11 $$ {\sum}_{\mathrm{j}}{\sum}_{\mathrm{i}}{\left({\mathrm{x}}_{\mathrm{i}\mathrm{j}}-{\mathrm{y}}_{\mathrm{i}\mathrm{j}}\right)}^2 $$

where x_ij_ is the predicted value and y_ij_ is the observed value of the fruit of the j^th^ banana plant at date t_i_.

### Goodness of fit and predictive quality of the model

The goodness of fit of the model was evaluated using two commonly used criteria, the root mean squared error (RMSE) and the relative root mean squared error (RRMSE), to compare the mean difference between simulated and observed results [37]. The smaller the value of RMSE and RRMSE, the better the fit.

12 $$ \mathrm{RMSE}=\surd \left({\displaystyle \sum {\left({\mathrm{y}}_{\mathrm{ij}}-{\mathrm{x}}_{\mathrm{ij}}\right)}^2/\mathrm{n}}\right) $$

where y_ij_ is the predicted value and x_ij_ is the measured value of the fruit of the j^th^ banana plant at date t_i_. n is the data number.

13 $$ \mathrm{RRMSE}=\mathrm{RMSE}/\overline{\mathrm{x}} $$

Where $\overline{\mathrm{x}}$ is the mean of all observed values.

The predictive quality of the model, which ascertains model validity over various scenarios, was quantified by the RMSE and RRMSE calculated using the 2012 data set (Additional file 6).

### Sensitivity analysis of the model

The sensitivity of the malate model during banana growth and post-harvest ripening to variations in parameter and input values was quantified by normalized sensitivity coefficients, defined as the ratio between the variation in malate concentration (ΔM) relative to its standard value (M), and the variation in the parameter or input value (ΔP) relative to its standard value (P) [38].

14 $$ \mathrm{Normalized}\ \mathrm{sensitivity}\ \mathrm{coefficient}=\left(\Delta \mathrm{M}/\mathrm{M}\right)/\left(\Delta \mathrm{P}/\mathrm{P}\right) $$

The interpretation of the sensitivity coefficient is referred to as local sensitivity analysis since these coefficients provide information on the effect of small changes in the parameters on the model response. They do not provide information about the effect of simultaneous or large parameter changes. Normalized sensitivity coefficients were calculated by altering one parameter or input variable by ±0.1% while keeping all other parameters and inputs at their default values. Sensitivity analysis of the model to parameters was conducted by considering pH_vac_ as known (approximated by the measured pH of the pulp). Sensitivity analysis of the model to pulp composition and temperature was conducted by considering the total model, i.e. the combination of the malate and pH models.

## Results

### Overview of the effects of the cultivar and of the treatment

The effects of cultivar and treatments on malate concentration in banana pulp during the pre and post-harvest stages are detailed in a previous paper [19], so only the main conclusions are presented here. During banana growth, the concentration of malate increased and was significantly affected by the cultivar in both 2011 and 2012. During banana post-harvest ripening, the ripening stage and the cultivar had a significant effect on the concentrations of malate in 2011 and 2012. Fruits harvested later (at 90% of FYT) had significantly higher concentrations of malate at the beginning of ripening and lower concentrations at the end of ripening. Low fruit load and potassium fertilization significantly increased fruit fresh mass but had no effect on malate concentration in the three cultivars either during growth or post-harvest ripening.

### Model calibration and evaluation

Values of the estimated parameters of the model are summarized in Table 1. The values of ΔG_ATP_ estimated during banana growth were higher (less negative) than the values commonly found in the literature, which range between −50 and −58 KJ mol^−1^ [31,32,39,40]. The ΔG_ATP_ estimated for the PL cultivar was lower (more negative) than those estimated for the IDN and PJB cultivars. During postharvest ripening, values of ΔG_ATP_ calculated from equation 10 with the estimated values of parameters G_1_, G_2_, and G_3_ were in the range of values found in the literature (between −45 and −55 KJ mol^−1^) (data not shown). From day 6 to the end of ripening, cultivars PJB and PL had a lower (more negative) ΔG_ATP_ than cultivar IDN.

**Figure 3:**
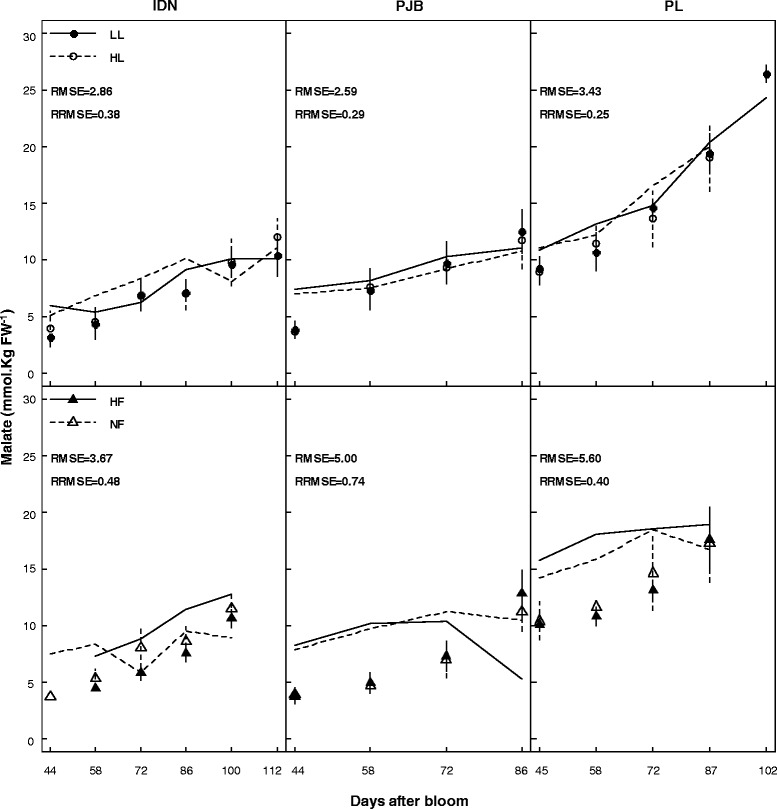
**Measured (symbols) and simulated (lines) malate concentrations in the pulp of banana of cultivars IDN, PJB, and PL during fruit growth.** The cultivars were grown under two contrasted fruit loads in 2011 (LL: low fruit load; HL: high fruit load), and two contrasted levels of potassium fertilization in 2012 (NF: no potassium fertilization; HF: high potassium fertilization). Data are means ± s.d (*n* = 6). The RMSE (mmol 100 g FW^−1^) and RRMSE are indicated in each graph.

**Figure 4:**
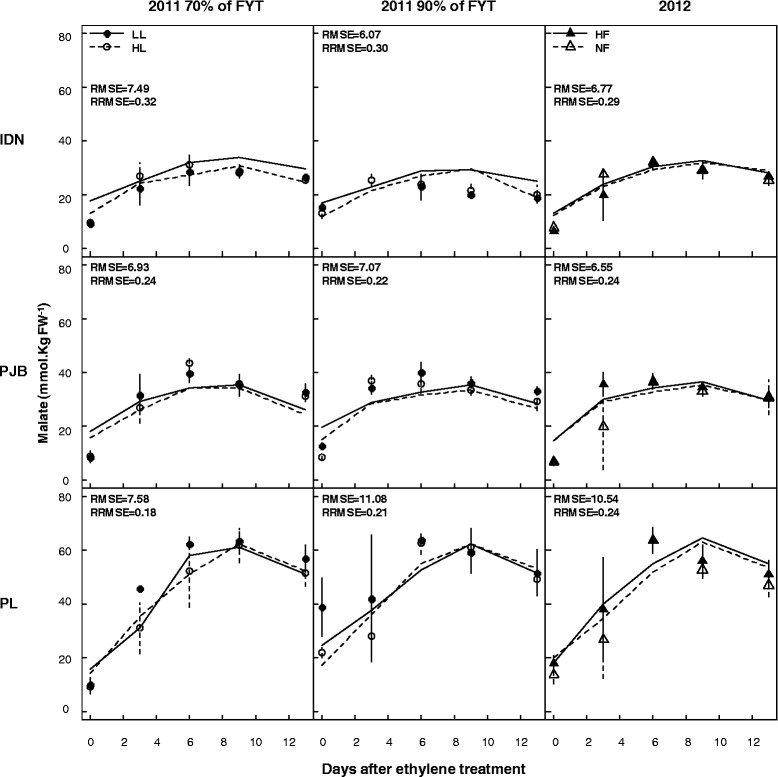
**Measured (symbols) and simulated (lines) malate concentrations in the pulp of banana of cultivars IDN, PJB, and PL during fruit post-harvest ripening**. The cultivars were grown under two contrasted fruit loads in 2011 (LL: low fruit load; HL: high fruit load), and two contrasted levels of potassium fertilization in 2012 (NF: no potassium fertilization; HF: high potassium fertilization). In 2011, fruits were harvested at two different stages: early stage (70% of FYT) and late stage (90% of FYT). Data are means ± s.d (*n* = 6). The RMSE (mmol 100 g FW^−1^) and RRMSE are indicated in each graph.

Simulated and observed malate concentrations during banana growth and post-harvest ripening are presented in Figures 3 and 4 respectively. For the three cultivars, the goodness of fit of predictions of data from 2011 was satisfactory both during banana growth and post-harvest ripening. During growth, the RMSEs were between 2.86 and 3.43 mmol Kg FW^−1^, and RRMSEs between 0.25 and 0.38. During postharvest ripening, the RMSEs were between 6.07 and 11.08 mmol Kg FW^−1^, and RRMSEs between 0.18 and 0.32. However, model validation during banana growth was not satisfactory in any of the three cultivars, as revealed by the RMSEs and RRMSEs of predictions of data from 2012, whose values ranged between 3.67 and 5.60 mmol Kg FW^−1^, and between 0.40 and 0.74 respectively. Model validation during banana post-harvest ripening for the three cultivars was satisfactory, as revealed by the RMSEs and RRMSEs of predictions of data from 2012, whose values ranged between 6.55 and 10.54 mmol Kg FW^−1^, and between 0.24 and 0.29 respectively. Statistical analysis revealed that the model predicted a large effect of the cultivar and of fruit age, and no effect of the fruit load and potassium fertilization on malate concentration during banana growth (Table 2) and postharvest ripening (Table 3) which is in accordance with observed data. The model predicted a small effect of fruit age at harvest in agreement with observed data, but was not able to simulate the minor differences correctly (data not shown).

**Table 2:** **LMM analysis of predicted and measured concentrations of malate (mmol Kg FW** ^**−1**^ **) during fruit growth**

**F-value** ^**a**^ **and significance** ^**b**^			
**Year**	**Factors** ^**c**^	**Predicted malate concentration**	**Measured malate concentration**
2011			
	c	51***	79***
	p	Ns	Ns
	a	78***	1599***
	a^2^	Ns	44***
	a^3^	Ns	9**
	p : a	Ns	Ns
	c : a	10***	155***
	c : p	Ns	Ns
	c : p : a	Ns	Ns
2012			
	c	77***	92***
	f	Ns	Ns
	a	8**	560***
	a^2^	7**	70***
	a^3^	5*	6**
	c : a	Ns	54***
	c : f	Ns	Ns
	f : a	Ns	Ns
	c : f : a	Ns	Ns

^a^The F-value is given only for the factors kept in the optimal model.

^b^***p-value <0.001; **p-value <0.01; *p-value < 0.05; Ns : not significant.

^c^Codes for factors: c = cultivar; p = pruning treatment; a = fruit age (in% of flowering-to-yellowing time); f = potassium fertilization treatment.

The factors studied were fruit age, cultivar, and pruning treatment in the 2011 experiment, and fruit age, cultivar, and potassium fertilization in the 2012 experiment. There were six replicates per combination cultivar∗treatment. Linear mixed-effects models [LMMs [41]] were used to examine the relationship between malate concentration and explanatory variables (fruit age, cultivar, treatment), and interactions. We used quadratic and cubic terms of fruit age when the curve passed through a maximum and had an asymmetrical shape. We used the lme function in the ‘nlme’ library [42] in the statistical program R 2.14.0. “Banana plant” was treated as a random effect because banana plants were assumed to contain unobserved heterogeneity, which is impossible to model. A temporal correlation structure was used to account for temporal pseudo-replication. Model selection was made using the top-down strategy [43]: starting with a model in which the fixed component contains all the explanatory variables and interactions, we found the optimal structure of the random component. We then used the F-statistic obtained with restricted maximum likelihood (REML) estimation to find the optimal fixed structure. Finally, the significance of each factor kept in the optimal model was assessed using the F-statistic obtained with REML estimation.

**Table 3:** **LMM analysis of predicted and measured malate concentration (mmol Kg FW** ^**−1**^ **) during post-harvest fruit ripening**

**F-value** ^**a**^ **and significance** ^**b**^			
**Year**	**Factors**	**Predicted malate concentration**	**Measured malate concentration**
2011			
	c	199***	284***
	p	Ns	Ns
	a	6*	11**
	r	363***	327***
	r^2^	563***	241***
	r^3^	12***	Ns
	p : r	Ns	Ns
	a : c	4*	15***
	a : r	Ns	15***
	c : r	92***	50***
	p : a	Ns	Ns
	p : c	Ns	Ns
	a:c:r	Ns	Ns
	p : a : c	Ns	Ns
	p : a : r	Ns	Ns
	p : a : c : r	Ns	Ns
2012			
	c	139***	73***
	f	Ns	Ns
	r	473***	386***
	r^2^	341***	184***
	r^3^	Ns	Ns
	c : f	Ns	Ns
	c : r	46***	51***
	f : r	Ns	Ns
	c : f : r	Ns	Ns

^a^The F-value is given only for the factors retained from the optimal model.

^b^*** p-value <0.001; **p-value <0.01; *p-value < 0.05; Ns: not significant.

^c^Codes for factors: c = cultivar; p = pruning treatment; a = fruit age at harvest; r = ripening stage; f = potassium fertilization treatment.

The factors studied were ripening stage, fruit age at harvest, cultivars, and pruning treatment in the 2011 experiment, and ripening stage, cultivars, and potassium fertilization treatment in the 2012 experiment.

### Sensitivity analysis of the model

**Figure 5:**
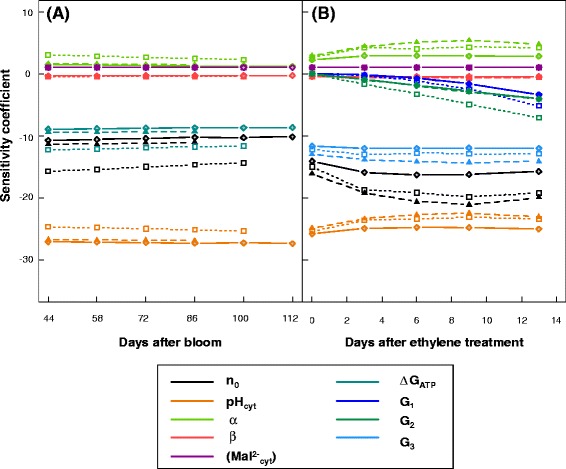
**Normalized sensitivity coefficients of the parameters of the malate model. (A)** Change in SCs during banana growth, and **(B)** post-harvest ripening for cultivar IDN (gray diamonds), PJB (black triangles), and PL (white squares).

A sensitivity coefficient (SC) was calculated to identify model responses to variations in parameters and inputs. A positive and negative sign of SC correspond, respectively, to a response in the same or reverse direction as the variation in the parameter or input. The larger the absolute value of SC, the more highly sensitive the model is to the parameter or input concerned. Since the SC behaved similarly between years with respect to a given cultivar, only results in 2011 are presented here. The SCs of model parameters behaved similarly with respect to the three cultivars and between banana growth (Figure 5A) and post-harvest ripening (Figure 5B). (Mal^2−^_cyt_) had a positive effect on malate concentration. This is as expected, since an increase in (Mal^2−^_cyt_) increases the gradient of concentration of the di-anion malate in favor of its transport into the vacuole. Malate concentration was greatly influenced by pH_cyt_ in a negative way. Malate accumulation decreases when cytosolic pH increases because the gradient of pH across the tonoplast increases, which depresses the ΔΨ (see equation 7). Increasing ΔG_ATP_, i.e. a less negative ΔG_ATP_, (which means increasing G_1_, G_2_, or G_3_ during postharvest ripening) depressed malate concentration, because it decreased proton pumping and consequently the ΔΨ. The parameter n_0_ had a strong negative effect on malate accumulation. This is as expected, since increasing n_0_ decreases the ΔΨ. The sensitivity to α was positive because increasing α increases the ΔΨ. The sensitivity to β was negative because increasing β decreases the ΔΨ.

**Figure 6:**
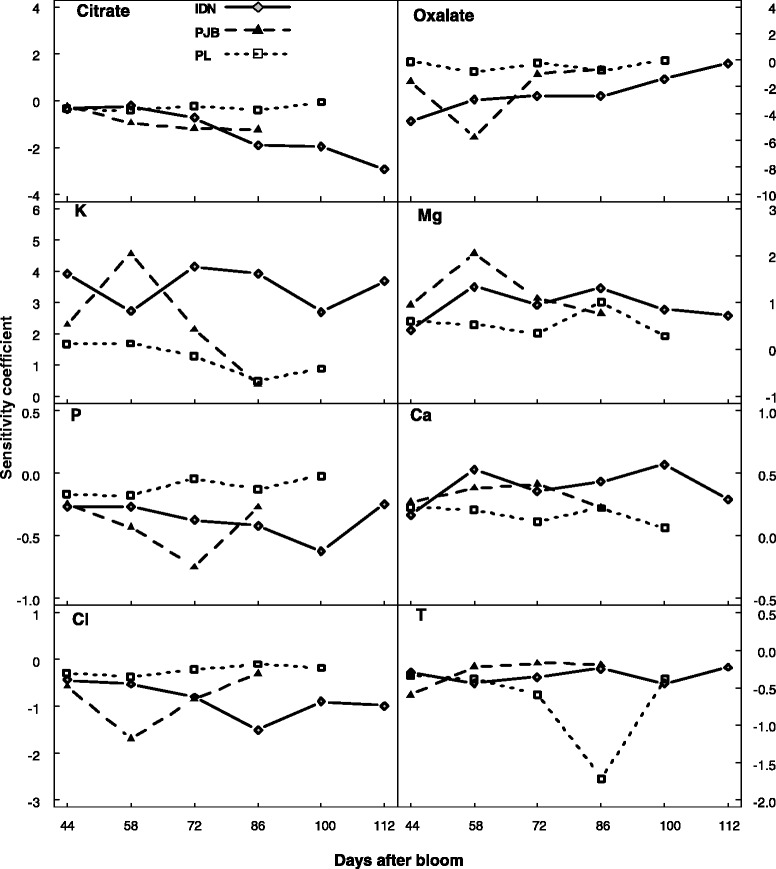
**Normalized sensitivity coefficients of the concentrations of citrate, oxalate, potassium (K), magnesium (Mg), phosphorus (P), calcium (Ca), and chloride (Cl) in the pulp, and of temperature (T) during banana growth for cultivars IDN, PJB, and PL.**

**Figure 7:**
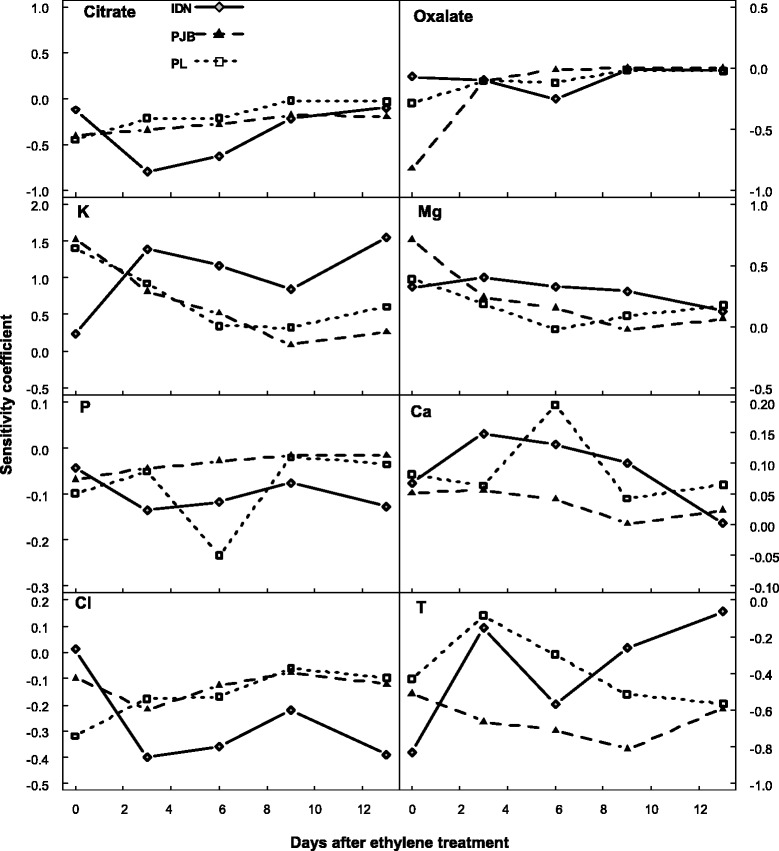
**Normalized sensitivity coefficients of the concentrations of citrate, oxalate, potassium (K), magnesium (Mg), phosphorus (P), calcium (Ca), and chloride (Cl) in the pulp, and of temperature (T) during banana post-harvest ripening for cultivars IDN, PJB, and PL.**

The SCs of model inputs during banana growth and post-harvest ripening are shown in Figures 6 and 7 respectively. Increasing citrate and oxalate concentration strongly depressed malate concentration during banana growth in all three cultivars. During postharvest ripening, citrate and oxalate concentration also had a negative but less important effect on malate concentration. Increasing K concentration had a strong positive effect on malate concentration during growth and a lesser effect during post-harvest ripening in the three cultivars. Increasing P concentration slightly depressed malate concentration both during growth and post-harvest ripening in the three cultivars. Increasing the Mg concentration had a positive effect on malate concentration during growth and a lesser effect during post-harvest ripening in all three cultivars. Increasing the Ca concentration had a slight positive effect on malate concentration both during growth and post-harvest ripening in all three cultivars. Increasing the Cl concentration had a negative effect on malate concentration during banana growth, and a lesser effect during post-harvest ripening in all three cultivars. Increasing temperature depressed malate accumulation during banana growth and post-harvest ripening in all three cultivars.

## Discussion

### Quality of predictions and model simplifications

**Figure 8:**
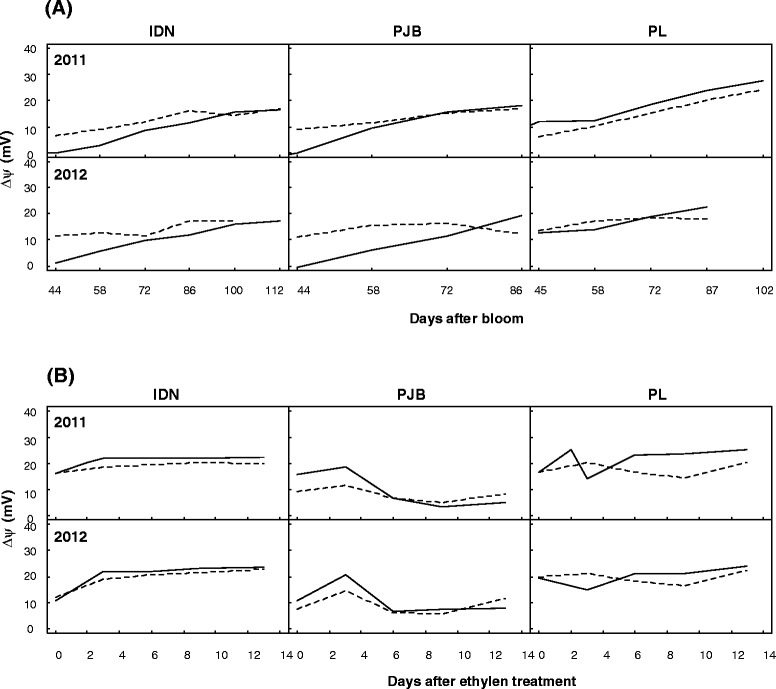
**Changes in Δψ during fruit development calculated from equation** 6 **(solid line) and from equation** 7 **(dashed line).** Values of Δψ were calculated for cultivars IDN, PJB, and PL in 2011 and 2012 during **(A)** banana growth, and **(B)** post-harvest ripening.

The concentrations of malate in the pulp were satisfactorily simulated by the model during postharvest ripening in the two experimental years, whereas model validation during fruit growth was not convincing. Differences in prediction quality between the pre and post-harvest stages have several possible explanations. First, the pH model was less accurate during fruit growth than during post-harvest ripening [18] which is certainly partially responsible for the discrepancies between observed and predicted malate concentrations during fruit growth. Second, we assumed that the ΔΨ was determined only by the ATPase functioning, whereas in reality, ΔΨ may also depend on the transport of mineral ions across the tonoplast (which generate currents and/or proton movements) and on the contribution of the PPiase to proton pumping [13]. To check if this hypothesis is reasonable, we compared the ΔΨ required to reach the thermodynamic equilibrium of the di-anion malate across the tonoplast (by inverting equation 6) with the ΔΨ predicted by the ATPase model (by inverting equation 7). During postharvest ripening, the changes in both ΔΨ were very similar (Figure 8B). Therefore, the ATPase model appears to be adequate for post-harvest ripening. This is consistent with the fact that at this stage, when mineral concentrations in the pulp remain constant [18], there should be no transport of minerals across the tonoplast. In addition, PPiase activity should be negligible since starch synthesis, which leads to the synthesis of PPi [27], has stopped. During fruit growth, there were some discrepancies between the variations in the ΔΨ calculated with equations 6 and 7, especially for cultivars IDN and PJB, for which malate predictions were worst (Figure 8A) and the ATPase model overestimated the ΔΨ required to sustain malate accumulation. During fruit growth, minerals, especially potassium, accumulate in the vacuole of pulp cells [18], which implies that electric currents may alter the ΔΨ. Moreover, starch synthesis is high, so that PPi might be available in large quantities and PPiase activity might consequently be important [27], however, to our knowledge, no information is available concerning the tonoplastic PPiase of banana fruit cells. In the future, predictions of malate concentrations during fruit growth might be improved by taking into account mineral fluxes and the possible contribution of the PPiase. Third, we assumed that pH_cyt_ and (Mal^2−^_cyt_) remained constant during fruit development, whereas in reality they certainly fluctuate in response to the supply of acids and bases by the sap, their metabolism, and their vacuolar storage. Since the model was very sensitive to cytosolic pH, one way to improve model predictions during fruit growth would be to take these possible fluctuations into account.

### Predicted variability in vacuolar malate accumulation among cultivars and between pre and post-harvest stages

The model revealed possible differences in vacuolar malate accumulation among the three cultivars studied here. During fruit growth, the value of estimated ΔG_ATP_ was a lot more negative for cultivar PL than for the two other cultivars, suggesting that the higher concentrations of malate in the fruits of cultivar PL could be the result of higher proton pumping due to a higher energy of ATP hydrolysis. During post-harvest ripening, the model predicted a more negative ΔG_ATP_ after ethylene treatment than before. This could be linked to the climacteric crisis. Indeed, the dramatic increase in respiration in response to ethylene treatment might be associated with an enhanced level of ATP exceeding the needs of the cells [44]. Consequently, the ratio of ATP to ADP might increase greatly, making ΔG_ATP_ more negative, which would increase the activity of the proton pumps and the accumulation of malate. The predicted increase in the activity of the proton pumps during banana ripening is in agreement with the results of Terrier *et al*. [45] on grape berry. The slight decrease in malate concentration at the end of ripening may be the consequence of a higher rate of malate leakage across the tonoplast, as observed in grape [45]. However, since this phenomenon was not represented in the present model, it resulted in a less negative ΔG_ATP_ at the end of ripening. The model predicted a significantly less negative ΔG_ATP_ for cultivar IDN than for cultivars PL and PJB, suggesting that the lower concentrations of malate in cultivar IDN might be due to lower proton pump activity. Differences in malate accumulation between cultivars PL and PJB were not due to differences in ΔG_ATP_, but to differences in vacuolar pH. Indeed, cultivar PL had a higher vacuolar pH than cultivar PJB during post-harvest ripening [18]. Vacuolar pH has contrasting effects on proton pump activity and malate dissociation. On one hand, increasing vacuolar pH decreases the di-anion concentration gradient, which reduces malate accumulation. On the other hand, it activates the proton pumps, which increases the ΔΨ and consequently malate accumulation. Finally, the positive effect on proton pump activity appears to prevail over the negative effect on malate dissociation. The possible involvement of vacuolar proton pumps in the difference in acidity among cultivars has been reported in peach [46] and in apple [7]. It should be noted that even though we assumed a common value of (Mal^2−^_cyt_) among cultivars, this parameter might be cultivar dependant, which would explain some of the differences in malate concentrations among cultivars. However, when we tried to fit the model with a common value of ΔG_ATP_ but different values of (Mal^2−^_cyt_) for the three cultivars, predictions were not in good agreement with the data (data not shown). This supports a role for ΔG_ATP_ in genotypic differences in malate accumulation.

### Model behavior

The positive effect of potassium concentration on malate accumulation revealed by the sensitivity analysis is in agreement with the positive relationships found in ripe peaches between malate content and ash alkalinity, which is closely linked with potassium content [47,48]. The model did not predict any effect of potassium fertilization on malate concentration, which is in agreement with observed data and with the fact that no significant differences in potassium concentration in banana pulp were found between the two treatments [19]. From a physiological point of view, increasing potassium concentration increases vacuolar pH (data not shown), which, according to the model, activates malate transport into the vacuole (see section Predicted variability in vacuolar malate accumulation among cultivars and between pre and post-harvest stages). According to the model, magnesium and chloride concentrations can influence malate accumulation, especially during fruit growth. Until now, no experiments have been conducted on the effects these minerals have on banana acidity, so it would be interesting to check the model predictions experimentally. The negative effect of organic acids (citrate and oxalate) on malate accumulation is the consequence of the decrease in vacuolar pH (see section Predicted variability in vacuolar malate accumulation among cultivars and between pre and post-harvest stages). The negative effect of temperature on the concentration of malate predicted by the model is in agreement with the results of Lobit *et al*. [17], and with some observations made in fields experiments on grape [49-51], and banana [52]. This is an interesting outcome of the model since temperature can easily be adjusted during post-harvest ripening. However, this result needs to be checked experimentally in post-harvest conditions.

### Model validity

The model was based on the hypothesis that malate di-anion and proton transport across the tonoplast occurs in conditions that are close to their respective thermodynamic equilibrium. We can see if these hypotheses are reasonable by checking that a number of conditions are met. One condition is that the ∆Ψ calculated under the assumption of the model falls within the range expected from data cited in the literature. We found that the ΔΨ calculated with the equation of the thermodynamic equilibrium of the di-anion malate across the tonoplast (equation 6) or with the ATPase model (equation 7) was between 0 and 25 mV (Figure 8), i.e. comparable with the expected ∆Ψ, which most authors estimate to be around 30 mV [53]. Therefore, the electric conditions of the vacuole appear to be compatible with the partitioning of the malate di-anion across the tonoplast in a state of thermodynamic equilibrium, and also with ATPase functioning in a state of thermodynamic equilibrium. Another condition is that the malate channel and the ATPase are not saturated; otherwise the transport of malate and proton would be limited by kinetic considerations and not just by thermodynamic considerations. In other words, the observed rate of malate accumulation must be lower than the maximum rate of malate transport through the di-anion channel, and the observed rate of proton accumulation must be lower than the maximum rate of proton transport through the ATPase. Concerning the malate channel, from the literature, Lobit *et al*. [17] calculated a maximum rate of malate transport of around 20 mmol jour^−1^ Kg FW^−1^. From our data, it can be calculated that the maximum rate of malate accumulation during banana development was 15 mmol jour^−1^ Kg FW^−1^. Therefore, the assumption that the activity of the malate transport system does not limit its storage appears to be reasonable. Concerning ATPase, from the literature, Lobit *et al*. [17] calculated a maximum rate of proton transport of around 50 mmol jour^−1^ Kg FW^−1^. From our data on titratable acidity [18], it can be calculated that the maximum rate of proton accumulation during banana development was 27 mmol jour^−1^ Kg FW^−1^. Therefore, the assumption that the activity of the ATPase does not limit proton pumping appears to be reasonable.

## Conclusion

In conclusion, the model proposed in this study predicted the concentration of malate in banana pulp during post-harvest ripening with good accuracy for three cultivars. However, it needs to be improved to predict malate concentration during fruit growth, maybe by taking into account the transport of minerals across the tonoplast, and/or the contribution of the PPiase, and/or possible fluctuations in cytosolic pH. The model suggested that the significant increase in malate concentration observed after the climacteric crisis could be due to an increase in ATPase activity in response to a higher free energy of ATP hydrolysis. The model also helped to dissect differences in malate accumulation among cultivars by highlighting the likely importance of the free energy of ATP hydrolysis and vacuolar pH. In the future, connecting such a model with a model of citrate prediction, and models relating titratable acidity and pulp composition [18], would provide a useful tool to study fruit acidity with an integrative view. Finally, the present adaptation of the malate model initially developed on peach, to banana fruit, highlights the possible generic quality of the model and its suitability for studying the genotypic variability and environmental regulation of malate accumulation in fleshy fruits during the pre and postharvest stages.

### Availability of supporting data

All the data supporting our results are included in the article and in the Additional files.

## Additional files

Additional file 1:
**A text file of the pre-harvest data of the 2011 experiment used to run the malate model.**
Additional file 2:
**A text file of the pre-harvest data of the 2012 experiment used to run the malate model.**
Additional file 3:
**A text file of the post-harvest data of the 2011 experiment used to run the malate model.**
Additional file 4:
**A text file of the post-harvest data of the 2012 experiment used to run the malate model.**
Additional file 5:
**The R scripts of the malate model.**
Additional file 6:
**An Excel spreadsheet containing the pre- and post-harvest data of the 2011 and 2012 experiments used for model calibration and validation.**

## Abbreviations

Ca: Pulp calcium content
Cl: Pulp chloride content
DAE: Day after ethylene treatment
DW: Pulp dry weight
FYT: Flowering-to-yellowing time
FW: Pulp fresh weight
IDN: Indonesia 110
K: Pulp potassium content
Mg: Pulp magnesium content
P: Pulp phosphorus content
PBSMs: Ecophysiological process-based simulation models
PJB: Pisang Jari Buaya
PL: Pisang Lilin
RMSE: Root mean squared error
RRMSE: Relative root mean squared error
SC: Sensitivity coefficient
T: Temperature
