# Supplementary material for: Modeling the vacuolar storage of malate shed lights on pre- and post-harvest fruit acidity
Source: BMC Plant Biol. 2014 Nov 18;14:310. doi: 10.1186/s12870-014-0310-7 (PMC4239383; doi:10.1186/s12870-014-0310-7)
Supplement: Additional file 5: — The R scripts of the malate model. [file 12870_2014_310_MOESM5_ESM.docx]

**SCRIPTS OF THE MODEL OF MALATE STORAGE**

**ALGORITHM OF THE MODEL**

This model uses the pH model well described in our paper: ***Etienne et al., 2013. A model approach revealed the relationship between banana pulp acidity and composition during growth and postharvest ripening***. Thus, for a detail explanation of this model, please refer to this paper.

h=[H^+^]=10^-x^=10^-pH^

x=pH_vac_=pH

All the elements are expressed as a function of h (or x) et μ=ionic strength

Calculation of [Mal_fruit_] ≅ [Mal_vac_] = f_Mal_(pH,μ) = f_Mal_(x,μ)

Calculation of apparent constants of acidity K'_i_

Calculation of the concentrations of the anions

[Cit^---^]=f_Cit3_(h,μ), [HCit^--^]=f_Cit2_(h,μ),…….

Calculation of the ionic strength μ=½'(9[Cit^---^]+…

**F_1_(μ,h)=0**

Calculation of the ionic balance

**F_2_(μ,h)=0**

Solving the following nonlinear system **F_1_(μ,h)=0**

(2 equations, 2 unknowns) **F_2_(μ,h)=0**

Calculation of [Mal_fruit_] ≅ [Mal_vac_]

**R SCRIPT OF THE MODEL OF MALATE STORAGE DURING BANANA GROWTH**

**######### Function "cmal" to calculate the concentration of malate in the vacuole**

**### Notations used in this function**

# T: temperature (Kelvin)

# a2Malcyt: Activity of the dianion malate in the cytosol (mol/L)

# Coeffa2vac: Activity coefficient of the dianion malate in the vacuole (Dimensionless)

# KK1; KK2: Apparent acidity constants of malate (mol/L)

# pHcyt: cytosolic pH (pH unit)

# pHvac: vacuolar pH (pH unit)

# i: Ionic strength in the vacuole (mol/L)

# FF: Constant of Faraday (96500 C/mol)

# R: Gas constant (8.314 J/mol/K)

# DY: Electric potential gradient across the tonoplast (Volts)

# cMalvac: Concentration of malate in the vacuole (mol/L)

# DGATP: Free energy of ATP hydrolysis (J/mol)

# n0; alpha; bbeta: Parameters used for the calculation of the ATPase stoechiometry (n) (Dimensionless)

**### Function**

cmal<-function(DGATP, T, n0, pHvac, alpha, bbeta, i, pHcyt, a2Malcyt, KK1, KK2)

{

R<-8.314 ; FF<-96500

# Calculation of the activity coefficient of the dianion malate in the vacuole (equation 4 in the paper on the pH model)

coeffa2vac<-10^(-0.5*4*((i^0.5/(1+i^0.5)-0.2*i)))

# Calculation of the electric potential gradient across the tonoplast (equation 7 of this paper)

DY<-(-DGATP)/(FF*(n0+alpha*(pHvac-7)+bbeta*10^(pHcyt-7)))+((R*T)/FF)*log(10)*(pHvac-pHcyt)

# Calculation of the concentration of malate in the vacuole (equation 6 of this paper)

cMalvac<-(1/coeffa2vac)* a2Malcyt * exp((2*FF*DY)/(R*T)) * (KK1*KK2+KK1*10^(-pHvac)+10^(-2*pHvac))/(KK1*KK2)

# Vector of outputs of the function : DY et cMalvac

c(DY,cMalvac)

}

**######## Function "acide" that calculates the pH and the ionic strength of the vacuole and then the concentration of malate in the vacuole**

**### Notations used in this function**

# Cit: Concentration of citrate in the pulp (mol/L)

# Oxa: Concentration of oxalate in the pulp (mol/L)

# Pho: Concentration of phosphorus in the pulp (mol/L)

# K: Concentration of potassium in the pulp (mol/L)

# Cl: Concentration of chloride in the pulp (mol/L)

# Mg: Concentration of magnesium in the pulp (mol/L)

# Ca: Concentration of calcium in the pulp (mol/L)

# Km1, Km2: Acidity constants of malate (mol/L)

# Kc1, Kc2, Kc3: Acidity constants of citrate (mol/L)

# Kp1, Kp2, Kp3: Acidity constants of phosphoric acid (mol/L)

# Ko1, Ko2: Acidity constants of oxalate (mol/L)

# h: 10-pH vacuole

# a0, a1, a2, a3: Activity coefficients of the different forms of the acids (Dimensionless)

# Ka1Mal, Ka2Mal: Apparent acidity constants of malate (mol/L)

# Ka1Cit, …: Apparent acidity constants of citrate (mol/L)

# Ka1PO, …: Apparent acidity constants of phosphoric acid (mol/L)

# Ka1Ox, …: Apparent acidity constants of oxalate (mol/L)

# mu: Ionic strength in the vacuole (mol/L)

# This function uses the function dsnlex to solve the system and calculate the pH and the ionic strength

library(nleqslv) # library to load

acide<-function(Cit,Oxa,Pho,K,Cl,Mg,Ca,DGATP, T, n0,alpha,bbeta,pHcyt,a2Malcyt)

**{**

# Acidity constants

Km1<-10^(-3.40) ; Km2<-10^(-5.11) # malate

Kc1<-10^(-3.14) ; Kc2<-10^(-4.77) ; Kc3<-10^(-6.39) # citrate

Kp1<-10^(-2.12) ; Kp2<-10^(-7.21) ; Kp3<-10^(-12.67) # phosphate

Ko1<-10^(-1.23) ; Ko2<-10^(-4.19) # oxalate

# the function « dslnex » is used by the « nleqslv » function to solve the system. This function takes as argument “x” which is the vector of the unknowns of the system. In the present case, **x[1] is pHvac and x[2] is the ionic strength of the vacuole**.

dslnex <- function(x)

**{**

**## Functions that calculates the proportion of each form of acid using the dissociation equations (equation 7 in the paper on the pH model)**

**# malate**

# tMal2 is the proportion of Mal^2-^: tMal2 =[Mal2-]/[Maltotal] (dimensionless)

# tMal1 is the proportion of HMal^-^

# tMal0 is the proportion of H_2_Mal

pMal2<-function(x, Km1, Km2) {

h<-10^(-x[2]) ; tMal2<-Km1*Km2/(h^2*(1+Km1/h+Km1*Km2/h^2))

tMal2} # the pMal2 function gives tMal2 as output

pMal1<- function(x, Km1, Km2) {

h <-10^(-x[2]) ; tMal1<-Km1/(h*(1+Km1/h+Km1*Km2/h^2))

tMal1}

pMal0<-function(x, Km1, Km2) {

h <-10^(-x[2]) ; tMal0<-1/(1*(1+Km1/h+Km1*Km2/h^2))

tMal0}

**# citrate**

# tCit3 is the proportion of Cit^3-^

# tCit2 is the proportion of HCit^2-^

# tCit1 is the proportion of H_2_Cit^-^

# tCit0 is the proportion of H_3_Cit

pCit3<-function(x, Kc1, Kc2, Kc3) {

h <-10^(-x[2]) ; tCit3<-Kc1*Kc2*Kc3/(h^3*(1+Kc1/h+Kc1*Kc2/h^2+Kc1*Kc2*Kc3/h^3))

tCit3}

pCit2<-function(x, Kc1, Kc2, Kc3) {

h<-10^(-x[2]); tCit2<-Kc1*Kc2/(h^2*(1+Kc1/h+Kc1*Kc2/h^2+Kc1*Kc2*Kc3/h^3))

tCit2}

pCit1<-function(x, Kc1, Kc2, Kc3) {

h <-10^(-x[2]) ; tCit1<-Kc1/(h*(1+Kc1/h+Kc1*Kc2/h^2+Kc1*Kc2*Kc3/h^3))

tCit1}

pCit0<-function(x, Kc1, Kc2, Kc3) {

h<-10^(-x[2]) ; tCit0<-1/(1*(1+Kc1/h+Kc1*Kc2/h^2+Kc1*Kc2*Kc3/h^3))

tCit0}

**# phosphate**

# tPho3 is the proportion of PO_4_^3-^

# tPho2 is the proportion of HPO_4_^2-^

# tPho1 is the proportion of H_2_PO^-^_4_

# tPho0 is the proportion of H_3_PO_4_

pPho3<-function(x, Kp1, Kp2, Kp3) {

h<-10^(-x[2]) ; tPho3<-Kp1*Kp2*Kp3/(h^3*(1+Kp1/h+Kp1*Kp2/h^2+Kp1*Kp2*Kp3/h^3))

tPho3}

pPho2<-function(x, Kp1, Kp2, Kp3) {

h<-10^(-x[2]) ; tPho2<-Kp1*Kp2/(h^2*(1+Kp1/h+Kp1*Kp2/h^2+Kp1*Kp2*Kp3/h^3))

tPho2}

pPho1<-function(x, Kp1, Kp2, Kp3) {

h<-10^(-x[2]) ; tPho1<-Kp1/(h*(1+Kp1/h+Kp1*Kp2/h^2+Kp1*Kp2*Kp3/h^3))

tPho1}

pPho0<-function(x, Kp1, Kp2, Kp3) {

h<-10^(-x[2]) ; tPho0<-1/(1*(1+Kp1/h+Kp1*Kp2/h^2+Kp1*Kp2*Kp3/h^3))

tPho0}

**# oxalate**

# tOxa2 is the proportion of Oxa^2-^

# tOxa1 is the proportion of HOxa^-^

# tOxa0 is the proportion of H_2_Oxa

pOxa2<-function(x, Ko1, Ko2) {

h<-10^(-x[2]) ; tOxa2<-Ko1*Ko2/(h^2*(1+Ko1/h+Ko1*Ko2/h^2))

tOxa2}

pOxa1<- function(x, Ko1, Ko2) {

h <-10^(-x[2]) ; tOxa1<-Ko1/(h*(1+Ko1/h+Ko1*Ko2/h^2))

tOxa1}

pOxa0<-function(x, Ko1, Ko2) {

h <-10^(-x[2]) ; tOxa0<-1/(1*(1+Ko1/h+Ko1*Ko2/h^2))

tOxa0}

**## Calculation of the activity coefficients in the vacuole in function of the ionic strength x[1] (equation 5 in the paper on the pH model)**

# a0 = activity coefficient of the fully protonated forms of the acids

a0<-1

# a1 = activity coefficient of the mono-anions

a1<-10^( ((-0.5*x[1]^0.5)/(1+x[1]^0.5))- (0.2*x[1]) )

# a2 activity coefficient of the di-anions

a2<-10^( ((-0.5*4*x[1]^0.5)/(1+x[1]^0.5)) - (0.2*x[1]))

# a3 = activity coefficient of the tri-anions

a3<-10^( ((-0.5*9*x[1]^0.5)/(1+x[1]^0.5)) - (0.2*x[1]))

**## Calculation of the apparent constants of acidity (equation 3 in the paper on the pH model)**

Ka1Mal<-Km1*(a0/a1) ; Ka2Mal<-Km2*(a1/a2) # malate

Ka1Cit<-Kc1*(a0/a1) ; Ka2Cit<-Kc2*(a1/a2) ; Ka3Cit<-Kc3*(a2/a3) # citrate

Ka1PO<-Kp1*(a0/a1) ; Ka2PO<-Kp2*(a1/a2) ; Ka3PO<-Kp3*(a2/a3) # phosphate

Ka1OX<-Ko1*(a0/a1) ; Ka2OX<-Ko2*(a1/a2) # oxalate

**## Calculation of the proportions of the different forms of the acids in the vacuole**

# the concentrations are in mol/L !!

**# Malate**

# the total concentration of malate in the vacuole (Mal) is calculated with the function « cmal »

Mal<-cmal(DGATP, T, n0, pHvac=x[2], i=x[1], alpha, bbeta, pHcyt, a2Malcyt, KK1=Ka1Mal, KK2=Ka2Mal)[2] # [2] is the output cMalvac of the function cmal

# pEMal2 = proportion of Mal2- ; EMal2 = concentration of Mal2- (mol/L)

# here the functions pMal…take as argument the apparent constants of acidity

pEMal2<-pMal2(x,Ka1Mal,Ka2Mal) ; EMal2<-Mal*pEMal2

pEMal1<-pMal1(x,Ka1Mal,Ka2Mal) ; EMal1<-Mal*pEMal1

pEMal0<-pMal0(x,Ka1Mal,Ka2Mal) ; EMal0<-Mal*pEMal0

**# citrate**

# Cit : total concentration of citrate in the vacuole

pECit3<-pCit3(x,Ka1Cit, Ka2Cit,Ka3Cit) ; ECit3<-Cit*pECit3

pECit2<-pCit2(x,Ka1Cit, Ka2Cit,Ka3Cit) ; ECit2<-Cit*pECit2

pECit1<-pCit1(x,Ka1Cit, Ka2Cit,Ka3Cit) ; ECit1<-Cit*pECit1

pECit0<-pCit0(x,Ka1Cit, Ka2Cit,Ka3Cit) ; ECit0<-Cit*pECit0

**# phosphate**

# Pho: total concentration of phosphate in the vacuole

pEPho3<-pPho3(x,Ka1PO, Ka2PO, Ka3PO) ; EPho3<-Pho*pEPho3

pEPho2<-pPho2(x,Ka1PO, Ka2PO, Ka3PO) ; EPho2<-Pho*pEPho2

pEPho1<-pPho1(x,Ka1PO, Ka2PO, Ka3PO) ; EPho1<-Pho*pEPho1

pEPho0<-pPho0(x,Ka1PO, Ka2PO, Ka3PO) ; EPho0<-Pho*pEPho0

**# oxalate**

# Oxa: total concentration of oxalate in the vacuole

pEOxa2<-pOxa2(x,Ka1OX,Ka2OX) ; EOxa2<-Oxa*pEOxa2

pEOxa1<-pOxa1(x,Ka1OX,Ka2OX) ; EOxa1<-Oxa*pEOxa1

pEOxa0<-pOxa0(x,Ka1OX,Ka2OX) ; EOxa0<-Oxa*pEOxa0

**## calculation of the ionic strength in the vacuole : mu (equation 4 in the paper on the pH model)**

mu<-0.5*(9*(ECit3+EPho3)+4*(ECit2+EMal2+EPho2+EOxa2)+ECit1+EMal1+EOxa1+EPho1+10^(-x[2])+10^(x[2]-14)+K+Cl+(4*Mg)+(4*Ca))

**## calculation of the sum of anions in the vacuole**

SommeAnions<-Cit*(3*pECit3+2*pECit2+pECit1)+Mal*(2*pEMal2+pEMal1)+Pho*(3*pEPho3+2*pEPho2+pEPho1)+Oxa*(2*pEOxa2+pEOxa1)+10^(x[2]-14)+ Cl

**## system to solve (equation F1 and F2 in the paper on the pH model)**

y <- numeric(2)

y[1]<-mu-x[1] # F2

y[2] <-SommeAnions -(10^(-x[2])+ K + (2*Mg)+ (2*Ca)) # F1

y

**}**

xstart <-c(0.001,4) # initial values of the two unknowns

nleqslv(xstart, dslnex, control=list(btol=.01,xtol=0.01)) # function that solves the system

**## table of results** : dataframe containing four variables :

# malate : concentration of malate in the vacuole (mol/L)

# DPsi : electric potential gradient across the tonoplast (volts)

# pH : vacuolar pH

# mu : ionic strength in the vacuole

resul<-data.frame(**malate**=cmal(DGATP, T, n0, pHvac=nleqslv(xstart, dslnex, control=list(btol=.01,xtol=0.01))$x[2], i=nleqslv(xstart, dslnex, control=list(btol=.01,xtol=0.01))$x[1], alpha, bbeta, pHcyt, a2Malcyt,

KK1=10^(-3.40)*(1/10^( ((-0.5*(nleqslv(xstart, dslnex, control=list(btol=.01,xtol=0.01))$x[1])^0.5)/(1+ (nleqslv(xstart, dslnex, control=list(btol=.01,xtol=0.01))$x[1])^0.5))- (0.2*(nleqslv(xstart, dslnex, control=list(btol=.01,xtol=0.01))$x[1])) ) ) ,

KK2=10^(-5.11)* ( 10^( ((-0.5*(nleqslv(xstart, dslnex, control=list(btol=.01,xtol=0.01))$x[1])^0.5)/(1+ (nleqslv(xstart, dslnex, control=list(btol=.01,xtol=0.01))$x[1])^0.5))- (0.2*(nleqslv(xstart, dslnex, control=list(btol=.01,xtol=0.01))$x[1])) ) / 10^( ((-0.5*4*(nleqslv(xstart, dslnex, control=list(btol=.01,xtol=0.01))$x[1])^0.5)/(1+(nleqslv(xstart, dslnex, control=list(btol=.01,xtol=0.01))$x[1])^0.5)) - (0.2*nleqslv(xstart, dslnex, control=list(btol=.01,xtol=0.01))$x[1])) ))[2],

DPsi=cmal(DGATP, T, n0, pHvac=nleqslv(xstart, dslnex, control=list(btol=.01,xtol=0.01))$x[2], i=nleqslv(xstart, dslnex, control=list(btol=.01,xtol=0.01))$x[1], alpha, bbeta, pHcyt, a2Malcyt,

KK1=10^(-3.40)*(1/10^( ((-0.5*(nleqslv(xstart, dslnex, control=list(btol=.01,xtol=0.01))$x[1])^0.5)/(1+ (nleqslv(xstart, dslnex, control=list(btol=.01,xtol=0.01))$x[1])^0.5))- (0.2*(nleqslv(xstart, dslnex, control=list(btol=.01,xtol=0.01))$x[1])) ) ) ,

KK2=10^(-5.11)* ( 10^( ((-0.5*(nleqslv(xstart, dslnex, control=list(btol=.01,xtol=0.01))$x[1])^0.5)/(1+ (nleqslv(xstart, dslnex, control=list(btol=.01,xtol=0.01))$x[1])^0.5))- (0.2*(nleqslv(xstart, dslnex, control=list(btol=.01,xtol=0.01))$x[1])) ) / 10^( ((-0.5*4*(nleqslv(xstart, dslnex, control=list(btol=.01,xtol=0.01))$x[1])^0.5)/(1+(nleqslv(xstart, dslnex, control=list(btol=.01,xtol=0.01))$x[1])^0.5)) - (0.2*nleqslv(xstart, dslnex, control=list(btol=.01,xtol=0.01))$x[1])) ))[1], **pH**=nleqslv(xstart, dslnex, control=list(btol=.01,xtol=0.01))$x[2], **mu**=nleqslv(xstart, dslnex, control=list(btol=.01,xtol=0.01))$x[1])

resul

**}**

**############ Function « ACIDE » that runs the function « acide » in a loop to calculate the concentration of malate in the pulp for each data inputs**

**### Notations used in this function**

# CMalfruit: Concentration of malate in the pulp (mmol/Kg FW)

# DELTAGATP: Free energy of ATP hydrolysis (J/mol)

# FWpulp: Pulp fresh weight (g)

# DWpulp: Pulp dry weight (g)

ACIDE<-function(DELTAGATP)

**{**

for (i in 1:dim(cap)[1]) **{** # “cap” is the dataframe of data inputs

fil[i,]<-acide(Cit=cap$cit[i],Oxa=cap$oxa[i],K=cap$K[i],Pho=cap$pho[i],Cl=cap$Cl[i],Mg=cap$Mg[i],Ca=cap$Ca[i], T=cap$TK[i], DGATP=DELTAGATP, n0=4, alpha=0.3, bbeta=-0.12, a2Malcyt=0.001, pHcyt=7)

# calculation of the concentration of malate in the pulp predicted by the model (mmol/kg FW)

fil$CMalfruit[i]<- fil$Mal[i]*((cap$FWpulp[i]- cap$DWpulp[i]) /cap$FWpulp[i]) *1000

**}**

# output of the function : dataframe « Fil » containing the following variables: malate ; DPsi ; pH ; mu ; CMalfruit

fil

**}**

**############ To run the model on the growth data of the 2011 experiment**

# Import of the input data : this file contains for each fruit sampled, the concentrations of soluble minerals elements and acids in the pulp, and the air temperature.

# You need to replace the following path by the path where your data file is stored

setwd("C:/Users/Audrey/Desktop/CD_thèse/modélisation/modèle malate/faire tourner le modèle")

donnees<-read.table("growth_data_2011.txt",header=TRUE, sep="", dec=".")

# Unit conversion of the concentration of malate measured in the fruits in mmol/kg FW

donnees$malatemmolKgMF<-((donnees$malateMF/100)/134)*1000*1000

# Suppression of the NA (missing data)

donnees<-donnees[!is.na(donnees$citrateMF),]

donnees<-donnees[!is.na(donnees$malateMF),]

donnees<-donnees[!is.na(donnees$oxalateMF),]

donnees<-donnees[!is.na(donnees$chloreMF),]

donnees<-donnees[!is.na(donnees$PhosphoreMF),]

donnees<-donnees[!is.na(donnees$CaMF),]

donnees<-donnees[!is.na(donnees$MgMF),]

donnees<-donnees[!is.na(donnees$KMF),]

donnees<-donnees[!is.na(donnees$FWpulp),]

donnees<-donnees[!is.na(donnees$DWpulp),]

# Unit conversion of the mineral concentrations in g/100g FW (they are originally in mg/100g FW)

donnees$PhosphoreMF<-donnees$PhosphoreMF/1000

donnees$KMF<-donnees$KMF/1000

donnees$ChloreMF<-donnees$chloreMF/1000

donnees$MgMF<-donnees$MgMF/1000

donnees$CaMF<-donnees$CaMF/1000

# Unit conversion of the concentrations in mol/L

donnees$mal<-donnees$malateMF*10/134

donnees$cit<-donnees$citrateMF*10/192

donnees$pho<-donnees$PhosphoreMF*10/31

donnees$K<-donnees$KMF*10/39.1

donnees$Cl<-donnees$ChloreMF*10/35.45

donnees$Mg<-donnees$MgMF*10/24.3

donnees$Ca<-donnees$CaMF*10/40.1

donnees$oxa<-donnees$oxalateMF*10/90

# Subset file for each genotype

pl_sc_2011<-subset(donnees,genotype=="PL")

idn_sc_2011<-subset(donnees,genotype=="IDN")

jb_sc_2011<-subset(donnees,genotype=="JB")

**##### Predictions for IDN**

# input data of IDN

cap<-idn_sc_2011

# Creation of the dataframe that will contain the variables calculated by the model

fil<-data.frame(Mal=rep(NA,dim(cap)[1]),DPSI=rep(NA,dim(cap)[1]), pHvac=rep(NA,dim(cap)[1]),mu=rep(NA,dim(cap)[1]),CMalfruit=rep(NA,dim(cap)[1]))

# We run the function « ACIDE » with a given value of DELTAGATP and store the results in an object

res_idn_sc_2011<-ACIDE(DELTAGATP=-36875.87)

# We merge the predicted and observed data

Malsimidn_sc_2011<-cbind(idn_sc_2011,res_idn_sc_2011)

**##### Predictions for PJB**

cap<-jb_sc_2011

fil<-data.frame(Mal=rep(NA,dim(cap)[1]),DPSI=rep(NA,dim(cap)[1]), pHvac=rep(NA,dim(cap)[1]),mu=rep(NA,dim(cap)[1]),CMalfruit=rep(NA,dim(cap)[1]))

res_jb_sc_2011<-ACIDE(DELTAGATP=-39108.69)

Malsimjb_sc_2011<-cbind(jb_sc_2011,res_jb_sc_2011)

**##### Predictions for PL**

cap<-pl_sc_2011

fil<-data.frame(Mal=rep(NA,dim(cap)[1]),DPSI=rep(NA,dim(cap)[1]), pHvac=rep(NA,dim(cap)[1]),mu=rep(NA,dim(cap)[1]),CMalfruit=rep(NA,dim(cap)[1]))

res_pl_sc_2011<-ACIDE(DELTAGATP=-47368.45)

Malsimpl_sc_2011<-cbind(pl_sc_2011,res_pl_sc_2011)

**############ To run the model on the growth data of the 2012 experiment**

# Import of the input data : this file contains for each fruit sampled, the concentrations of soluble minerals elements and acids in the pulp, and the air temperature.

don_sc_ferti<-read.table("growth_data_2012.txt",header=TRUE, sep="", dec=".")

# Unit conversion of the concentration of malate measured in the fruits in mmol/kg FW

don_sc_ferti$malatemmolKgMF<-((don_sc_ferti$malateMF/100)/134)*1000*1000

# Suppression of the NA (missing data)

don_sc_ferti<-don_sc_ferti[!is.na(don_sc_ferti$citrateMF),]

don_sc_ferti<-don_sc_ferti[!is.na(don_sc_ferti$malateMF),]

don_sc_ferti<-don_sc_ferti[!is.na(don_sc_ferti$oxalateMF),]

don_sc_ferti<-don_sc_ferti[!is.na(don_sc_ferti$chloreMF),]

don_sc_ferti<-don_sc_ferti[!is.na(don_sc_ferti$PhosphoreMF),]

don_sc_ferti<-don_sc_ferti[!is.na(don_sc_ferti$CaMF),]

don_sc_ferti<-don_sc_ferti[!is.na(don_sc_ferti$MgMF),]

don_sc_ferti<-don_sc_ferti[!is.na(don_sc_ferti$KMF),]

don_sc_ferti<-don_sc_ferti[!is.na(don_sc_ferti$PFpulpe),]

don_sc_ferti<-don_sc_ferti[!is.na(don_sc_ferti$PSpulpe),]

# Unit conversion of the mineral concentrations in g/100g FW (they are originally in mg/100g FW)

don_sc_ferti$PhosphoreMF<-don_sc_ferti$PhosphoreMF/1000

don_sc_ferti$KMF<-don_sc_ferti$KMF/1000

don_sc_ferti$ChloreMF<-don_sc_ferti$chloreMF/1000

don_sc_ferti$MgMF<-don_sc_ferti$MgMF/1000

don_sc_ferti$CaMF<-don_sc_ferti$CaMF/1000

# Unit conversion of the concentrations in mol/L

don_sc_ferti$mal<-don_sc_ferti$malateMF*10/134

don_sc_ferti$cit<-don_sc_ferti$citrateMF*10/192

don_sc_ferti$pho<-don_sc_ferti$PhosphoreMF*10/31

don_sc_ferti$K<-don_sc_ferti$KMF*10/39.1

don_sc_ferti$Cl<-don_sc_ferti$ChloreMF*10/35.45

don_sc_ferti$Mg<-don_sc_ferti$MgMF*10/24.3

don_sc_ferti$Ca<-don_sc_ferti$CaMF*10/40.1

don_sc_ferti$oxa<-don_sc_ferti$oxalateMF*10/90

# Subset file for each genotype

pl_sc_ferti<-subset(don_sc_ferti,genotype=="PL")

idn_sc_ferti<-subset(don_sc_ferti,genotype=="IDN")

jb_sc_ferti<-subset(don_sc_ferti,genotype=="JB")

**##### Predictions for IDN**

cap<-idn_sc_ferti

fil<-data.frame(Mal=rep(NA,dim(cap)[1]),DPSI=rep(NA,dim(cap)[1]), pHvac=rep(NA,dim(cap)[1]),mu=rep(NA,dim(cap)[1]),CMalfruit=rep(NA,dim(cap)[1]))

res_idn_sc_ferti<-ACIDE(DELTAGATP=-36875.87)

Malsimidn_sc_ferti<-cbind(idn_sc_ferti,res_idn_sc_ferti)

**##### Predictions for PJB**

cap<-jb_sc_ferti

fil<-data.frame(Mal=rep(NA,dim(cap)[1]),DPSI=rep(NA,dim(cap)[1]), pHvac=rep(NA,dim(cap)[1]),mu=rep(NA,dim(cap)[1]),CMalfruit=rep(NA,dim(cap)[1]))

res_jb_sc_ferti<-ACIDE(DELTAGATP=-39108.69)

Malsimjb_sc_ferti<-cbind(jb_sc_ferti,res_jb_sc_ferti)

**##### Predictions for PL**

cap<-pl_sc_ferti

fil<-data.frame(Mal=rep(NA,dim(cap)[1]),DPSI=rep(NA,dim(cap)[1]), pHvac=rep(NA,dim(cap)[1]),mu=rep(NA,dim(cap)[1]),CMalfruit=rep(NA,dim(cap)[1]))

res_pl_sc_ferti<-ACIDE(DELTAGATP=-47368.45)

Malsimpl_sc_ferti<-cbind(pl_sc_ferti,res_pl_sc_ferti)

**R SCRIPT OF THE MODEL OF MALATE STORAGE DURING BANANA POSTHARVEST RIPENING**

**######### Function "cmal" to calculate the concentration of malate in the vacuole**

**### Notations used in this function**

# T: temperature (Kelvin)

# a2Malcyt: Activity of the dianion malate in the cytosol (mol/L)

# Coeffa2vac: Activity coefficient of the dianion malate in the vacuole (Dimensionless)

# KK1; KK2: Apparent acidity constants of malate (mol/L)

# pHcyt: cytosolic pH (pH unit)

# pHvac: vacuolar pH (pH unit)

# i: Ionic strength in the vacuole (mol/L)

# FF: Constant of Faraday (96500 C/mol)

# R: Gas constant (8.314 J/mol/K)

# DY: Electric potential gradient across the tonoplast (Volts)

# cMalvac: Concentration of malate in the vacuole (mol/L)

# DGATP: Free energy of ATP hydrolysis (J/mol)

# n0; alpha; bbeta: Parameters used for the calculation of the ATPase stoechiometry (n) (Dimensionless)

# m1, m2, m3: parameters used for the calculation of DGATP

**### Function**

cmal<-function(m1,m2,m3,jour,T, n0, pHvac, alpha, bbeta, i, pHcyt, a2Malcyt, KK1, KK2)

{

R<-8.314 ; FF<-96500

coeffa2vac<-10^(-0.5*4*((i^0.5/(1+i^0.5)-0.2*i)))

DGATP<-m1*jour^2+m2*jour+m3

DY<-(-DGATP)/(FF*(n0+alpha*(pHvac-7)+bbeta*10^(pHcyt-7)))+((R*T)/FF)*log(10)*(pHvac-pHcyt)

cMalvac<-(1/coeffa2vac)* a2Malcyt * exp((2*FF*DY)/(R*T)) * (KK1*KK2+KK1*10^(-pHvac)+10^(-2*pHvac))/(KK1*KK2)

c(DY,cMalvac,DGATP)

}

**######## Function "acide" that calculates the pH and the ionic strength of the vacuole and then the concentration of malate in the vacuole**

**### Notations used in this function**

# Cit: Concentration of citrate in the pulp (mol/L)

# Oxa: Concentration of oxalate in the pulp (mol/L)

# Pho: Concentration of phosphorus in the pulp (mol/L)

# K: Concentration of potassium in the pulp (mol/L)

# Cl: Concentration of chloride in the pulp (mol/L)

# Mg: Concentration of magnesium in the pulp (mol/L)

# Ca: Concentration of calcium in the pulp (mol/L)

# Km1, Km2: Acidity constants of malate (mol/L)

# Kc1, Kc2, Kc3: Acidity constants of citrate (mol/L)

# Kp1, Kp2, Kp3: Acidity constants of phosphoric acid (mol/L)

# Ko1, Ko2: Acidity constants of oxalate (mol/L)

# h: 10-pH vacuole

# a0, a1, a2, a3: Activity coefficients of the different forms of the acids (Dimensionless)

# Ka1Mal, Ka2Mal: Apparent acidity constants of malate (mol/L)

# Ka1Cit, …: Apparent acidity constants of citrate (mol/L)

# Ka1PO, …: Apparent acidity constants of phosphoric acid (mol/L)

# Ka1Ox, …: Apparent acidity constants of oxalate (mol/L)

# mu: Ionic strength in the vacuole (mol/L)

# m1, m2, m3: parameters used for the calculation of DGATP

# This function uses the function dsnlex to solve the system and calculate the pH and the ionic strength

library(nleqslv) # library to load

acide<-function(Cit,Oxa,Pho,K,Cl,Mg,Ca,m1,m2,m3,jour, T, n0,alpha,bbeta,pHcyt,a2Malcyt)

{

# Acidity constants

Km1<-10^(-3.40) ; Km2<-10^(-5.11) # malate

Kc1<-10^(-3.14) ; Kc2<-10^(-4.77) ; Kc3<-10^(-6.39) # citrate

Kp1<-10^(-2.12) ; Kp2<-10^(-7.21) ; Kp3<-10^(-12.67) # phosphate

Ko1<-10^(-1.23) ; Ko2<-10^(-4.19) # oxalate

dslnex <- function(x)

{

**## Functions that calculates the proportion of each form of acid using the dissociation equations (equation 7 in the paper on the pH model)**

**# malate**

pMal2<-function(x, Km1, Km2) {

h<-10^(-x[2]) ; tMal2<-Km1*Km2/(h^2*(1+Km1/h+Km1*Km2/h^2))

tMal2}

pMal1<- function(x, Km1, Km2) {

h <-10^(-x[2]) ; tMal1<-Km1/(h*(1+Km1/h+Km1*Km2/h^2))

tMal1}

pMal0<-function(x, Km1, Km2) {

h <-10^(-x[2]) ; tMal0<-1/(1*(1+Km1/h+Km1*Km2/h^2))

tMal0}

**# citrate**

pCit3<-function(x, Kc1, Kc2, Kc3) {

h <-10^(-x[2]) ; tCit3<-Kc1*Kc2*Kc3/(h^3*(1+Kc1/h+Kc1*Kc2/h^2+Kc1*Kc2*Kc3/h^3))

tCit3}

pCit2<-function(x, Kc1, Kc2, Kc3) {

h<-10^(-x[2]); tCit2<-Kc1*Kc2/(h^2*(1+Kc1/h+Kc1*Kc2/h^2+Kc1*Kc2*Kc3/h^3))

tCit2}

pCit1<-function(x, Kc1, Kc2, Kc3) {

h <-10^(-x[2]) ; tCit1<-Kc1/(h*(1+Kc1/h+Kc1*Kc2/h^2+Kc1*Kc2*Kc3/h^3))

tCit1}

pCit0<-function(x, Kc1, Kc2, Kc3) {

h<-10^(-x[2]) ; tCit0<-1/(1*(1+Kc1/h+Kc1*Kc2/h^2+Kc1*Kc2*Kc3/h^3))

tCit0}

**# phosphate**

pPho3<-function(x, Kp1, Kp2, Kp3) {

h<-10^(-x[2]) ; tPho3<-Kp1*Kp2*Kp3/(h^3*(1+Kp1/h+Kp1*Kp2/h^2+Kp1*Kp2*Kp3/h^3))

tPho3}

pPho2<-function(x, Kp1, Kp2, Kp3) {

h<-10^(-x[2]) ; tPho2<-Kp1*Kp2/(h^2*(1+Kp1/h+Kp1*Kp2/h^2+Kp1*Kp2*Kp3/h^3))

tPho2}

pPho1<-function(x, Kp1, Kp2, Kp3) {

h<-10^(-x[2]) ; tPho1<-Kp1/(h*(1+Kp1/h+Kp1*Kp2/h^2+Kp1*Kp2*Kp3/h^3))

tPho1}

pPho0<-function(x, Kp1, Kp2, Kp3) {

h<-10^(-x[2]) ; tPho0<-1/(1*(1+Kp1/h+Kp1*Kp2/h^2+Kp1*Kp2*Kp3/h^3))

tPho0}

**# oxalate**

pOxa2<-function(x, Ko1, Ko2) {

h<-10^(-x[2]) ; tOxa2<-Ko1*Ko2/(h^2*(1+Ko1/h+Ko1*Ko2/h^2))

tOxa2}

pOxa1<- function(x, Ko1, Ko2) {

h <-10^(-x[2]) ; tOxa1<-Ko1/(h*(1+Ko1/h+Ko1*Ko2/h^2))

tOxa1}

pOxa0<-function(x, Ko1, Ko2) {

h <-10^(-x[2]) ; tOxa0<-1/(1*(1+Ko1/h+Ko1*Ko2/h^2))

tOxa0}

**## Calculation of the activity coefficients in the vacuole in function of the ionic strength x[1] (equation 5 in the paper on the pH model)**

# a0 = activity coefficient of the fully protonated forms of the acids

a0<-1

# a1 = activity coefficient of the mono-anions

a1<-10^( ((-0.5*x[1]^0.5)/(1+x[1]^0.5))- (0.2*x[1]) )

# a2 activity coefficient of the di-anions

a2<-10^( ((-0.5*4*x[1]^0.5)/(1+x[1]^0.5)) - (0.2*x[1]))

# a3 = activity coefficient of the tri-anions

a3<-10^( ((-0.5*9*x[1]^0.5)/(1+x[1]^0.5)) - (0.2*x[1]))

**## Calculation of the apparent constants of acidity (equation 3 in the paper on the pH model)**

Ka1Mal<-Km1*(a0/a1) ; Ka2Mal<-Km2*(a1/a2) # malate

Ka1Cit<-Kc1*(a0/a1) ; Ka2Cit<-Kc2*(a1/a2) ; Ka3Cit<-Kc3*(a2/a3) # citrate

Ka1PO<-Kp1*(a0/a1) ; Ka2PO<-Kp2*(a1/a2) ; Ka3PO<-Kp3*(a2/a3) # phosphate

Ka1OX<-Ko1*(a0/a1) ; Ka2OX<-Ko2*(a1/a2) # oxalate

**## Calculation of the proportions of the different forms of the acids in the vacuole**

**# Malate**

# the total concentration of malate in the vacuole (Mal) is calculated with the function « cmal »

Mal<-cmal(m1,m2,m3,jour, T, n0, pHvac=x[2], i=x[1], alpha, bbeta, pHcyt, a2Malcyt, KK1=Ka1Mal, KK2=Ka2Mal)[2]

pEMal2<-pMal2(x,Ka1Mal,Ka2Mal) ; EMal2<-Mal*pEMal2

pEMal1<-pMal1(x,Ka1Mal,Ka2Mal) ; EMal1<-Mal*pEMal1

pEMal0<-pMal0(x,Ka1Mal,Ka2Mal) ; EMal0<-Mal*pEMal0

**# citrate**

# Cit : total concentration of citrate in the vacuole

pECit3<-pCit3(x,Ka1Cit, Ka2Cit,Ka3Cit) ; ECit3<-Cit*pECit3

pECit2<-pCit2(x,Ka1Cit, Ka2Cit,Ka3Cit) ; ECit2<-Cit*pECit2

pECit1<-pCit1(x,Ka1Cit, Ka2Cit,Ka3Cit) ; ECit1<-Cit*pECit1

pECit0<-pCit0(x,Ka1Cit, Ka2Cit,Ka3Cit) ; ECit0<-Cit*pECit0

**# phosphate**

# Pho: total concentration of phosphate in the vacuole

pEPho3<-pPho3(x,Ka1PO, Ka2PO, Ka3PO) ; EPho3<-Pho*pEPho3

pEPho2<-pPho2(x,Ka1PO, Ka2PO, Ka3PO) ; EPho2<-Pho*pEPho2

pEPho1<-pPho1(x,Ka1PO, Ka2PO, Ka3PO) ; EPho1<-Pho*pEPho1

pEPho0<-pPho0(x,Ka1PO, Ka2PO, Ka3PO) ; EPho0<-Pho*pEPho0

**# oxalate**

# Oxa: total concentration of oxalate in the vacuole

pEOxa2<-pOxa2(x,Ka1OX,Ka2OX) ; EOxa2<-Oxa*pEOxa2

pEOxa1<-pOxa1(x,Ka1OX,Ka2OX) ; EOxa1<-Oxa*pEOxa1

pEOxa0<-pOxa0(x,Ka1OX,Ka2OX) ; EOxa0<-Oxa*pEOxa0

**## calculation of the ionic strength in the vacuole : mu (equation 4 in the paper on the pH model)**

mu<-0.5*(9*(ECit3+EPho3)+4*(ECit2+EMal2+EPho2+EOxa2)+ECit1+EMal1+EOxa1+EPho1+10^(-x[2])+10^(x[2]-14)+K+Cl+(4*Mg)+(4*Ca))

**## calculation of the sum of anions in the vacuole**

SommeAnions<-Cit*(3*pECit3+2*pECit2+pECit1)+Mal*(2*pEMal2+pEMal1)+Pho*(3*pEPho3+2*pEPho2+pEPho1)+Oxa*(2*pEOxa2+pEOxa1)+10^(x[2]-14)+ Cl

**## system to solve (equation F1 and F2 in the paper on the pH model)**

y <- numeric(2)

y[1]<-mu-x[1] # F1

y[2] <-SommeAnions -(10^(-x[2])+ K + (2*Mg)+ (2*Ca)) # F2

y

}

xstart <-c(0.001,4) # initial values of the two unknowns

nleqslv(xstart, dslnex, control=list(btol=.01,xtol=0.01)) # function that solves the system

**## table of results** : dataframe containing five variables :

# malate : concentration of malate in the vacuole (mol/L)

# DPsi : electric potential gradient across the tonoplast (volts)

# pH : vacuolar pH

# mu : ionic strength in the vacuole

# DGatp: free energy of ATP hydrolysis

resul<-data.frame(malate=cmal(m1,m2,m3,jour, T, n0, pHvac=nleqslv(xstart, dslnex, control=list(btol=.01,xtol=0.01))$x[2], i=nleqslv(xstart, dslnex, control=list(btol=.01,xtol=0.01))$x[1], alpha, bbeta, pHcyt, a2Malcyt,

KK1=10^(-3.40)*(1/10^( ((-0.5*(nleqslv(xstart, dslnex, control=list(btol=.01,xtol=0.01))$x[1])^0.5)/(1+ (nleqslv(xstart, dslnex, control=list(btol=.01,xtol=0.01))$x[1])^0.5))- (0.2*(nleqslv(xstart, dslnex, control=list(btol=.01,xtol=0.01))$x[1])) ) ) ,

KK2=10^(-5.11)* ( 10^( ((-0.5*(nleqslv(xstart, dslnex, control=list(btol=.01,xtol=0.01))$x[1])^0.5)/(1+ (nleqslv(xstart, dslnex, control=list(btol=.01,xtol=0.01))$x[1])^0.5))- (0.2*(nleqslv(xstart, dslnex, control=list(btol=.01,xtol=0.01))$x[1])) ) / 10^( ((-0.5*4*(nleqslv(xstart, dslnex, control=list(btol=.01,xtol=0.01))$x[1])^0.5)/(1+(nleqslv(xstart, dslnex, control=list(btol=.01,xtol=0.01))$x[1])^0.5)) - (0.2*nleqslv(xstart, dslnex, control=list(btol=.01,xtol=0.01))$x[1])) ))[2],

DPsi=cmal(m1,m2,m3,jour, T, n0, pHvac=nleqslv(xstart, dslnex, control=list(btol=.01,xtol=0.01))$x[2], i=nleqslv(xstart, dslnex, control=list(btol=.01,xtol=0.01))$x[1], alpha, bbeta, pHcyt, a2Malcyt,

KK1=10^(-3.40)*(1/10^( ((-0.5*(nleqslv(xstart, dslnex, control=list(btol=.01,xtol=0.01))$x[1])^0.5)/(1+ (nleqslv(xstart, dslnex, control=list(btol=.01,xtol=0.01))$x[1])^0.5))- (0.2*(nleqslv(xstart, dslnex, control=list(btol=.01,xtol=0.01))$x[1])) ) ) ,

KK2=10^(-5.11)* ( 10^( ((-0.5*(nleqslv(xstart, dslnex, control=list(btol=.01,xtol=0.01))$x[1])^0.5)/(1+ (nleqslv(xstart, dslnex, control=list(btol=.01,xtol=0.01))$x[1])^0.5))- (0.2*(nleqslv(xstart, dslnex, control=list(btol=.01,xtol=0.01))$x[1])) ) / 10^( ((-0.5*4*(nleqslv(xstart, dslnex, control=list(btol=.01,xtol=0.01))$x[1])^0.5)/(1+(nleqslv(xstart, dslnex, control=list(btol=.01,xtol=0.01))$x[1])^0.5)) - (0.2*nleqslv(xstart, dslnex, control=list(btol=.01,xtol=0.01))$x[1])) ))[1],pH=nleqslv(xstart, dslnex, control=list(btol=.01,xtol=0.01))$x[2], mu=nleqslv(xstart, dslnex, control=list(btol=.01,xtol=0.01))$x[1],

DGatp=cmal(m1,m2,m3,jour, T, n0, pHvac=nleqslv(xstart, dslnex, control=list(btol=.01,xtol=0.01))$x[2], i=nleqslv(xstart, dslnex, control=list(btol=.01,xtol=0.01))$x[1], alpha, bbeta, pHcyt, a2Malcyt,

KK1=10^(-3.40)*(1/10^( ((-0.5*(nleqslv(xstart, dslnex, control=list(btol=.01,xtol=0.01))$x[1])^0.5)/(1+ (nleqslv(xstart, dslnex, control=list(btol=.01,xtol=0.01))$x[1])^0.5))- (0.2*(nleqslv(xstart, dslnex, control=list(btol=.01,xtol=0.01))$x[1])) ) ) ,

KK2=10^(-5.11)* ( 10^( ((-0.5*(nleqslv(xstart, dslnex, control=list(btol=.01,xtol=0.01))$x[1])^0.5)/(1+ (nleqslv(xstart, dslnex, control=list(btol=.01,xtol=0.01))$x[1])^0.5))- (0.2*(nleqslv(xstart, dslnex, control=list(btol=.01,xtol=0.01))$x[1])) ) / 10^( ((-0.5*4*(nleqslv(xstart, dslnex, control=list(btol=.01,xtol=0.01))$x[1])^0.5)/(1+(nleqslv(xstart, dslnex, control=list(btol=.01,xtol=0.01))$x[1])^0.5)) - (0.2*nleqslv(xstart, dslnex, control=list(btol=.01,xtol=0.01))$x[1])) ))[3])

resul

}

**############ Function « ACIDE » that runs the function « acide » in a loop to calculate the concentration of malate in the pulp for each data inputs**

**### Notations used in this function**

# CMalfruit: Concentration of malate in the pulp (mmol/Kg FW)

# FWpulp: Pulp fresh weight (g)

# DWpulp: Pulp dry weight (g)

# M1, M2, M3: parameters to calculate DGatp

ACIDE<-function(M1,M2,M3)

{

for (i in 1:dim(cap)[1]) {

fil[i,]<-acide(Cit=cap$cit[i],Oxa=cap$oxa[i],K=cap$K[i],Pho=cap$pho[i],Cl=cap$Cl[i],Mg=cap$Mg[i],Ca=cap$Ca[i], jour=cap$stage[i], T=291.15, m1=M1, m2=M2, m3=M3,n0=4, alpha=0.3, bbeta=-0.12, a2Malcyt=0.001, pHcyt=7)

fil$CMalfruit[i]<- fil$Mal[i]*((cap$FWpulp[i]- cap$DWpulp[i]) /cap$FWpulp[i]) *1000

}

# output of the function : dataframe « Fil » containing the following variables: malate ; DPsi ; pH ; mu ; DGatp; CMalfruit

fil

}

**############ To run the model on the postharvest data of the 2011 experiment**

# Import of the input data : this file contains for each fruit sampled, the concentrations of soluble minerals elements and acids in the pulp, and the air temperature.

# You need to replace the following path by the path where your data file is stored

setwd("C:/Users/Audrey/Desktop/CD_thèse/modélisation/modèle malate/faire tourner le modèle")

donnees<-read.table("postharvest_data_2011.txt",header=TRUE, sep="", dec=".")

# Unit conversion of the concentration of malate measured in the fruits in mmol/kg FW

donnees$malatemmolKgMF<-((donnees$malateMF/100)/134)*1000*1000

# Suppression of the NA (missing data)

donnees<-donnees[!is.na(donnees$citrateMF),]

donnees<-donnees[!is.na(donnees$malateMF),]

donnees<-donnees[!is.na(donnees$oxalateMF),]

donnees<-donnees[!is.na(donnees$chloreMF),]

donnees<-donnees[!is.na(donnees$PhosphoreMF),]

donnees<-donnees[!is.na(donnees$CaMF),]

donnees<-donnees[!is.na(donnees$MgMF),]

donnees<-donnees[!is.na(donnees$KMF),]

donnees<-donnees[!is.na(donnees$FWpulp),]

donnees<-donnees[!is.na(donnees$DWpulp),]

# Unit conversion of the mineral concentrations in g/100g FW (they are originally in mg/100g FW)

donnees$PhosphoreMF<-donnees$PhosphoreMF/1000

donnees$KMF<-donnees$KMF/1000

donnees$ChloreMF<-donnees$chloreMF/1000

donnees$MgMF<-donnees$MgMF/1000

donnees$CaMF<-donnees$CaMF/1000

# Unit conversion of the concentrations in mol/L

donnees$mal<-donnees$malateMF*10/134

donnees$cit<-donnees$citrateMF*10/192

donnees$pho<-donnees$PhosphoreMF*10/31

donnees$K<-donnees$KMF*10/39.1

donnees$Cl<-donnees$ChloreMF*10/35.45

donnees$Mg<-donnees$MgMF*10/24.3

donnees$Ca<-donnees$CaMF*10/40.1

donnees$oxa<-donnees$oxalateMF*10/90

# Subset file for each genotype

pl_sm_2011<-subset(donnees,genotype=="PL")

idn_sm_2011<-subset(donnees,genotype=="IDN")

jb_sm_2011<-subset(donnees,genotype=="JB")

**##### Predictions for IDN**

cap<-idn_sm_2011

fil<-data.frame(Mal=rep(NA,dim(cap)[1]),DPSI=rep(NA,dim(cap)[1]), pHvac=rep(NA,dim(cap)[1]),mu=rep(NA,dim(cap)[1]),DGatp=rep(NA,dim(cap)[1]), CMalfruit=rep(NA,dim(cap)[1]))

res_idn_sm_2011<-ACIDE(M1=74.80396,M2=-1175.76452,M3=-45223.47823)

Malsimidn_sm_2011<-cbind(idn_sm_2011,res_idn_sm_2011)

**##### Predictions for PJB**

cap<-jb_sm_2011

fil<-data.frame(Mal=rep(NA,dim(cap)[1]),DPSI=rep(NA,dim(cap)[1]), pHvac=rep(NA,dim(cap)[1]),mu=rep(NA,dim(cap)[1]),DGatp=rep(NA,dim(cap)[1]), CMalfruit=rep(NA,dim(cap)[1]))

res_jb_sm_2011<-ACIDE(M1=68.77985,M2=-1108.34325,M3=-48950.81526)

Malsimjb_sm_2011<-cbind(jb_sm_2011,res_jb_sm_2011)

**##### Predictions for PL**

cap<-pl_sm_2011

fil<-data.frame(Mal=rep(NA,dim(cap)[1]),DPSI=rep(NA,dim(cap)[1]), pHvac=rep(NA,dim(cap)[1]),mu=rep(NA,dim(cap)[1]),DGatp=rep(NA,dim(cap)[1]), CMalfruit=rep(NA,dim(cap)[1]))

res_pl_sm_2011<-ACIDE(M1=109.7021,M2=-1959.3208,M3=-46289.7339)

Malsimpl_sm_2011<-cbind(pl_sm_2011,res_pl_sm_2011)

**############ To run the model on the postharvest data of the 2012 experiment**

# Import of the input data : this file contains for each fruit sampled, the concentrations of soluble minerals elements and acids in the pulp, and the air temperature.

don_sm_ferti<-read.table("postharvest_data_2012.txt",header=TRUE, sep="", dec=".")

# Unit conversion of the concentration of malate measured in the fruits in mmol/kg FW

don_sm_ferti$malatemmolKgMF<-((don_sm_ferti$malateMF/100)/134)*1000*1000

# Suppression of the NA (missing data)

don_sm_ferti<-don_sm_ferti[!is.na(don_sm_ferti$citrateMF),]

don_sm_ferti<-don_sm_ferti[!is.na(don_sm_ferti$malateMF),]

don_sm_ferti<-don_sm_ferti[!is.na(don_sm_ferti$oxalateMF),]

don_sm_ferti<-don_sm_ferti[!is.na(don_sm_ferti$chloreMF),]

don_sm_ferti<-don_sm_ferti[!is.na(don_sm_ferti$PhosphoreMF),]

don_sm_ferti<-don_sm_ferti[!is.na(don_sm_ferti$CaMF),]

don_sm_ferti<-don_sm_ferti[!is.na(don_sm_ferti$MgMF),]

don_sm_ferti<-don_sm_ferti[!is.na(don_sm_ferti$KMF),]

don_sm_ferti<-don_sm_ferti[!is.na(don_sm_ferti$FWpulp),]

don_sm_ferti<-don_sm_ferti[!is.na(don_sm_ferti$DWpulp),]

# Unit conversion of the mineral concentrations in g/100g FW (they are originally in mg/100g FW)

don_sm_ferti$PhosphoreMF<-don_sm_ferti$PhosphoreMF/1000

don_sm_ferti$KMF<-don_sm_ferti$KMF/1000

don_sm_ferti$ChloreMF<-don_sm_ferti$chloreMF/1000

don_sm_ferti$MgMF<-don_sm_ferti$MgMF/1000

don_sm_ferti$CaMF<-don_sm_ferti$CaMF/1000

# Unit conversion of the concentrations in mol/L

don_sm_ferti$mal<-don_sm_ferti$malateMF*10/134

don_sm_ferti$cit<-don_sm_ferti$citrateMF*10/192

don_sm_ferti$pho<-don_sm_ferti$PhosphoreMF*10/31

don_sm_ferti$K<-don_sm_ferti$KMF*10/39.1

don_sm_ferti$Cl<-don_sm_ferti$ChloreMF*10/35.45

don_sm_ferti$Mg<-don_sm_ferti$MgMF*10/24.3

don_sm_ferti$Ca<-don_sm_ferti$CaMF*10/40.1

don_sm_ferti$oxa<-don_sm_ferti$oxalateMF*10/90

# Subset file for each genotype

pl_sm_ferti<-subset(don_sm_ferti,genotype=="PL")

idn_sm_ferti<-subset(don_sm_ferti, genotype =="IDN")

jb_sm_ferti<-subset(don_sm_ferti, genotype =="JB")

**##### Predictions for IDN**

cap<-idn_sm_ferti

fil<-data.frame(Mal=rep(NA,dim(cap)[1]),DPSI=rep(NA,dim(cap)[1]), pHvac=rep(NA,dim(cap)[1]),mu=rep(NA,dim(cap)[1]),DGatp=rep(NA,dim(cap)[1]), CMalfruit=rep(NA,dim(cap)[1]))

res_idn_sm_ferti<-ACIDE(M1=74.80396,M2=-1175.76452,M3=-45223.47823)

Malsimidn_sm_ferti<-cbind(idn_sm_ferti,res_idn_sm_ferti)

**##### Predictions for PJB**

cap<-jb_sm_ferti

fil<-data.frame(Mal=rep(NA,dim(cap)[1]),DPSI=rep(NA,dim(cap)[1]), pHvac=rep(NA,dim(cap)[1]),mu=rep(NA,dim(cap)[1]),DGatp=rep(NA,dim(cap)[1]), CMalfruit=rep(NA,dim(cap)[1]))

res_jb_sm_ferti<-ACIDE(M1=68.77985,M2=-1108.34325,M3=-48950.81526)

Malsimjb_sm_ferti<-cbind(jb_sm_ferti,res_jb_sm_ferti)

**##### Predictions for PL**

cap<-pl_sm_ferti

# dataframe qui va contenir les valeurs calculées

fil<-data.frame(Mal=rep(NA,dim(cap)[1]),DPSI=rep(NA,dim(cap)[1]), pHvac=rep(NA,dim(cap)[1]),mu=rep(NA,dim(cap)[1]),DGatp=rep(NA,dim(cap)[1]), CMalfruit=rep(NA,dim(cap)[1]))

res_pl_sm_ferti<-ACIDE(M1=109.7021,M2=-1959.3208,M3=-46289.7339)

Malsimpl_sm_ferti<-cbind(pl_sm_ferti,res_pl_sm_ferti)

**R SCRIPT TO MAKE THE GRAPHICS OF OBSERVATIONS AND PREDICTIONS IN 2011 AND 2012 FOR THE POSTHARVEST STAGE**

# Suppression of aberrant predictions

Malsimjb_sm_2011<-Malsimjb_sm_2011[Malsimjb_sm_2011$CMalfruit<70,]

Malsimpl_sm_ferti<-Malsimpl_sm_ferti[Malsimpl_sm_ferti$CMalfruit<100,]

####### Calculation of the mean and sd of the predictions by genotype and by ripening stage

### IDN 2011

moy_idn_sm_2011<-aggregate(Malsimidn_sm_2011[,16:31],by=list(Malsimidn_sm_2011$SR,Malsimidn_sm_2011$treatment,Malsimidn_sm_2011$stage),FUN=mean)

ect_idn_sm_2011<-aggregate(Malsimidn_sm_2011[,16:31],by=list(Malsimidn_sm_2011$SR,Malsimidn_sm_2011$treatment,Malsimidn_sm_2011$stage),FUN=sd)

compil_idn_sm_2011<-merge(moy_idn_sm_2011,ect_idn_sm_2011,by=c("Group.1","Group.2","Group.3"))

### JB 2011

moy_jb_sm_2011<-aggregate(Malsimjb_sm_2011[,16:31],by=list(Malsimjb_sm_2011$SR,Malsimjb_sm_2011$treatment,Malsimjb_sm_2011$stage),FUN=mean)

ect_jb_sm_2011<-aggregate(Malsimjb_sm_2011[,16:31],by=list(Malsimjb_sm_2011$SR,Malsimjb_sm_2011$treatment,Malsimjb_sm_2011$stage),FUN=sd)

compil_jb_sm_2011<-merge(moy_jb_sm_2011,ect_jb_sm_2011,by=c("Group.1","Group.2","Group.3"))

### PL 2011

moy_pl_sm_2011<-aggregate(Malsimpl_sm_2011[,16:31],by=list(Malsimpl_sm_2011$SR,Malsimpl_sm_2011$treatment,Malsimpl_sm_2011$stage),FUN=mean)

ect_pl_sm_2011<-aggregate(Malsimpl_sm_2011[,16:31],by=list(Malsimpl_sm_2011$SR,Malsimpl_sm_2011$treatment,Malsimpl_sm_2011$stage),FUN=sd)

compil_pl_sm_2011<-merge(moy_pl_sm_2011,ect_pl_sm_2011,by=c("Group.1","Group.2","Group.3"))

### IDN 2012

moy_idn_sm_ferti<-aggregate(Malsimidn_sm_ferti[,15:30],by=list(Malsimidn_sm_ferti$treatment,Malsimidn_sm_ferti$stage),FUN=mean)

ect_idn_sm_ferti<-aggregate(Malsimidn_sm_ferti[,15:30],by=list(Malsimidn_sm_ferti$treatment,Malsimidn_sm_ferti$stage),FUN=sd)

compil_idn_sm_ferti<-merge(moy_idn_sm_ferti,ect_idn_sm_ferti,by=c("Group.1","Group.2"))

### JB 2012

moy_jb_sm_ferti<-aggregate(Malsimjb_sm_ferti[,15:30],by=list(Malsimjb_sm_ferti$treatment,Malsimjb_sm_ferti$stage),FUN=mean)

ect_jb_sm_ferti<-aggregate(Malsimjb_sm_ferti[,15:30],by=list(Malsimjb_sm_ferti$treatment,Malsimjb_sm_ferti$stage),FUN=sd)

compil_jb_sm_ferti<-merge(moy_jb_sm_ferti,ect_jb_sm_ferti,by=c("Group.1","Group.2"))

### PL 2012

moy_pl_sm_ferti<-aggregate(Malsimpl_sm_ferti[,15:30],by=list(Malsimpl_sm_ferti$treatment,Malsimpl_sm_ferti$stage),FUN=mean)

ect_pl_sm_ferti<-aggregate(Malsimpl_sm_ferti[,15:30],by=list(Malsimpl_sm_ferti$treatment,Malsimpl_sm_ferti$stage),FUN=sd)

compil_pl_sm_ferti<-merge(moy_pl_sm_ferti,ect_pl_sm_ferti,by=c("Group.1","Group.2"))

# to sort data by increasing growing stage (in order to have a nice line graphic)

compil_idn_sm_2011<-compil_idn_sm_2011[order(compil_idn_sm_2011$Group.1,compil_idn_sm_2011$Group.2,compil_idn_sm_2011$Group.3),]

compil_jb_sm_2011<-compil_jb_sm_2011[order(compil_jb_sm_2011$Group.1,compil_jb_sm_2011$Group.2,compil_jb_sm_2011$Group.3),]

compil_pl_sm_2011<-compil_pl_sm_2011[order(compil_pl_sm_2011$Group.1,compil_pl_sm_2011$Group.2,compil_pl_sm_2011$Group.3),]

compil_idn_sm_ferti<-compil_idn_sm_ferti[order(compil_idn_sm_ferti$Group.1,compil_idn_sm_ferti$Group.2),]

compil_jb_sm_ferti<-compil_jb_sm_ferti[order(compil_jb_sm_ferti$Group.1,compil_jb_sm_ferti$Group.2),]

compil_pl_sm_ferti<-compil_pl_sm_ferti[order(compil_pl_sm_ferti$Group.1,compil_pl_sm_ferti$Group.2),]

##### Drawing the graphics

layout(matrix(1:9, 3, 3))

layout.show(9)

## Harvest stage 70% of FYT in 2011

# IDN

par(mar=c(0,0,0,0))

plot(malatemmolKgMF.x~Group.3,data=compil_idn_sm_2011[compil_idn_sm_2011$Group.2=="A"&compil_idn_sm_2011$Group.1=="SM70",],ylim=c(0,80),xlim=c(0,13),xlab="stade",ylab="malate (mmol/Kg FW)",cex.lab=1.3,font.lab=2,pch=16,las=1,tck=0.02,xaxt="n",cex=1.5)

axis(side=1,tck=0.02,labels=FALSE)

points(malatemmolKgMF.x~Group.3,data=compil_idn_sm_2011[compil_idn_sm_2011$Group.2=="T"&compil_idn_sm_2011$Group.1=="SM70",],pch=1,cex=1.5)

points(CMalfruit.x~Group.3,data=compil_idn_sm_2011[compil_idn_sm_2011$Group.2=="A"&compil_idn_sm_2011$Group.1=="SM70",],type="l",lty=1,ljoin="round",lwd=1)

points(CMalfruit.x~Group.3,data=compil_idn_sm_2011[compil_idn_sm_2011$Group.2=="T"&compil_idn_sm_2011$Group.1=="SM70",],type="l",lty=2,lwd=1,ljoin="round")

# to draw the standard deviations

for (i in 1:5) {segments(compil_idn_sm_2011$Group.3[compil_idn_sm_2011$Group.2=="A"&compil_idn_sm_2011$Group.1=="SM70"][i],compil_idn_sm_2011$malatemmolKgMF.x[compil_idn_sm_2011$Group.2=="A"&compil_idn_sm_2011$Group.1=="SM70"][i]-compil_idn_sm_2011$malatemmolKgMF.y[compil_idn_sm_2011$Group.2=="A"&compil_idn_sm_2011$Group.1=="SM70"][i] ,compil_idn_sm_2011$Group.3[compil_idn_sm_2011$Group.2=="A"&compil_idn_sm_2011$Group.1=="SM70"][i],compil_idn_sm_2011$malatemmolKgMF.x[compil_idn_sm_2011$Group.2=="A"&compil_idn_sm_2011$Group.1=="SM70"][i]+compil_idn_sm_2011$malatemmolKgMF.y[compil_idn_sm_2011$Group.2=="A"&compil_idn_sm_2011$Group.1=="SM70"][i],lty=1)}

for (i in 1:5) {segments(compil_idn_sm_2011$Group.3[compil_idn_sm_2011$Group.2=="T"&compil_idn_sm_2011$Group.1=="SM70"][i],compil_idn_sm_2011$malatemmolKgMF.x[compil_idn_sm_2011$Group.2=="T"&compil_idn_sm_2011$Group.1=="SM70"][i]-compil_idn_sm_2011$malatemmolKgMF.y[compil_idn_sm_2011$Group.2=="T"&compil_idn_sm_2011$Group.1=="SM70"][i] ,compil_idn_sm_2011$Group.3[compil_idn_sm_2011$Group.2=="T"&compil_idn_sm_2011$Group.1=="SM70"][i],compil_idn_sm_2011$malatemmolKgMF.x[compil_idn_sm_2011$Group.2=="T"&compil_idn_sm_2011$Group.1=="SM70"][i]+compil_idn_sm_2011$malatemmolKgMF.y[compil_idn_sm_2011$Group.2=="T"&compil_idn_sm_2011$Group.1=="SM70"][i],lty=2)}

# to draw the legend

points(1,80,pch=16,cex=1.5)

segments(0,80,2,80,lty=1)

text(2.5,80,adj=0,"LL",cex=1,font=2)

points(1,75,pch=1,cex=1.5)

segments(0,75,2,75,lty=2)

text(2.5,75,adj=0,"HL",cex=1,font=2)

# JB

par(mar=c(0,0,0,0))

plot(malatemmolKgMF.x~Group.3,data=compil_jb_sm_2011[compil_jb_sm_2011$Group.2=="A"&compil_jb_sm_2011$Group.1=="SM70",],ylim=c(0,80),xlim=c(0,13),xlab="stade",ylab="malate (mmol/Kg FW)",cex.lab=1.3,font.lab=2,pch=16,las=1,tck=0.02,xaxt="n",cex=1.5,yaxt="n")

axis(side=1,tck=0.02,labels=FALSE)

axis(side=2,tck=0.02,labels=FALSE)

points(malatemmolKgMF.x~Group.3,data=compil_jb_sm_2011[compil_jb_sm_2011$Group.2=="T"&compil_jb_sm_2011$Group.1=="SM70",],pch=1,cex=1.5)

points(CMalfruit.x~Group.3,data=compil_jb_sm_2011[compil_jb_sm_2011$Group.2=="A"&compil_jb_sm_2011$Group.1=="SM70",],type="l",lty=1,ljoin="round",lwd=1)

points(CMalfruit.x~Group.3,data=compil_jb_sm_2011[compil_jb_sm_2011$Group.2=="T"&compil_jb_sm_2011$Group.1=="SM70",],type="l",lty=2,lwd=1,ljoin="round")

# to draw the standard deviations

for (i in 1:5) {segments(compil_jb_sm_2011$Group.3[compil_jb_sm_2011$Group.2=="A"&compil_jb_sm_2011$Group.1=="SM70"][i],compil_jb_sm_2011$malatemmolKgMF.x[compil_jb_sm_2011$Group.2=="A"&compil_jb_sm_2011$Group.1=="SM70"][i]-compil_jb_sm_2011$malatemmolKgMF.y[compil_jb_sm_2011$Group.2=="A"&compil_jb_sm_2011$Group.1=="SM70"][i] ,compil_jb_sm_2011$Group.3[compil_jb_sm_2011$Group.2=="A"&compil_jb_sm_2011$Group.1=="SM70"][i],compil_jb_sm_2011$malatemmolKgMF.x[compil_jb_sm_2011$Group.2=="A"&compil_jb_sm_2011$Group.1=="SM70"][i]+compil_jb_sm_2011$malatemmolKgMF.y[compil_jb_sm_2011$Group.2=="A"&compil_jb_sm_2011$Group.1=="SM70"][i],lty=1)}

for (i in 1:5) {segments(compil_jb_sm_2011$Group.3[compil_jb_sm_2011$Group.2=="T"&compil_jb_sm_2011$Group.1=="SM70"][i],compil_jb_sm_2011$malatemmolKgMF.x[compil_jb_sm_2011$Group.2=="T"&compil_jb_sm_2011$Group.1=="SM70"][i]-compil_jb_sm_2011$malatemmolKgMF.y[compil_jb_sm_2011$Group.2=="T"&compil_jb_sm_2011$Group.1=="SM70"][i] ,compil_jb_sm_2011$Group.3[compil_jb_sm_2011$Group.2=="T"&compil_jb_sm_2011$Group.1=="SM70"][i],compil_jb_sm_2011$malatemmolKgMF.x[compil_jb_sm_2011$Group.2=="T"&compil_jb_sm_2011$Group.1=="SM70"][i]+compil_jb_sm_2011$malatemmolKgMF.y[compil_jb_sm_2011$Group.2=="T"&compil_jb_sm_2011$Group.1=="SM70"][i],lty=2)}

# PL

par(mar=c(0,0,0,0))

plot(malatemmolKgMF.x~Group.3,data=compil_pl_sm_2011[compil_pl_sm_2011$Group.2=="A"&compil_pl_sm_2011$Group.1=="SM70",],ylim=c(0,80),xlim=c(0,13),xlab="stade",ylab="malate (mmol/Kg FW)",cex.lab=1.3,font.lab=2,pch=16,las=1,tck=0.02,xaxt="n",cex=1.5,yaxt="n")

axis(side=1,tck=0.02,labels=FALSE)

axis(side=2,tck=0.02,labels=FALSE)

points(malatemmolKgMF.x~Group.3,data=compil_pl_sm_2011[compil_pl_sm_2011$Group.2=="T"&compil_pl_sm_2011$Group.1=="SM70",],pch=1,cex=1.5)

points(CMalfruit.x~Group.3,data=compil_pl_sm_2011[compil_pl_sm_2011$Group.2=="A"&compil_pl_sm_2011$Group.1=="SM70",],type="l",lty=1,ljoin="round",lwd=1)

points(CMalfruit.x~Group.3,data=compil_pl_sm_2011[compil_pl_sm_2011$Group.2=="T"&compil_pl_sm_2011$Group.1=="SM70",],type="l",lty=2,lwd=1,ljoin="round")

# to draw the standard deviations

for (i in 1:5) {segments(compil_pl_sm_2011$Group.3[compil_pl_sm_2011$Group.2=="A"&compil_pl_sm_2011$Group.1=="SM70"][i],compil_pl_sm_2011$malatemmolKgMF.x[compil_pl_sm_2011$Group.2=="A"&compil_pl_sm_2011$Group.1=="SM70"][i]-compil_pl_sm_2011$malatemmolKgMF.y[compil_pl_sm_2011$Group.2=="A"&compil_pl_sm_2011$Group.1=="SM70"][i] ,compil_pl_sm_2011$Group.3[compil_pl_sm_2011$Group.2=="A"&compil_pl_sm_2011$Group.1=="SM70"][i],compil_pl_sm_2011$malatemmolKgMF.x[compil_pl_sm_2011$Group.2=="A"&compil_pl_sm_2011$Group.1=="SM70"][i]+compil_pl_sm_2011$malatemmolKgMF.y[compil_pl_sm_2011$Group.2=="A"&compil_pl_sm_2011$Group.1=="SM70"][i],lty=1)}

for (i in 1:5) {segments(compil_pl_sm_2011$Group.3[compil_pl_sm_2011$Group.2=="T"&compil_pl_sm_2011$Group.1=="SM70"][i],compil_pl_sm_2011$malatemmolKgMF.x[compil_pl_sm_2011$Group.2=="T"&compil_pl_sm_2011$Group.1=="SM70"][i]-compil_pl_sm_2011$malatemmolKgMF.y[compil_pl_sm_2011$Group.2=="T"&compil_pl_sm_2011$Group.1=="SM70"][i] ,compil_pl_sm_2011$Group.3[compil_pl_sm_2011$Group.2=="T"&compil_pl_sm_2011$Group.1=="SM70"][i],compil_pl_sm_2011$malatemmolKgMF.x[compil_pl_sm_2011$Group.2=="T"&compil_pl_sm_2011$Group.1=="SM70"][i]+compil_pl_sm_2011$malatemmolKgMF.y[compil_pl_sm_2011$Group.2=="T"&compil_pl_sm_2011$Group.1=="SM70"][i],lty=2)}

## Harvest stage 90% of FYT 2011

# IDN

par(mar=c(0,0,0,0))

plot(malatemmolKgMF.x~Group.3,data=compil_idn_sm_2011[compil_idn_sm_2011$Group.2=="A"&compil_idn_sm_2011$Group.1=="SM90",],ylim=c(0,80),xlim=c(0,13),xlab="stade",ylab="malate (mmol/Kg FW)",cex.lab=1.3,font.lab=2,pch=16,las=1,tck=0.02,xaxt="n",cex=1.5,yaxt="n")

axis(side=1,tck=0.02,labels=FALSE)

axis(side=2,tck=0.02,labels=FALSE)

points(malatemmolKgMF.x~Group.3,data=compil_idn_sm_2011[compil_idn_sm_2011$Group.2=="T"&compil_idn_sm_2011$Group.1=="SM90",],pch=1,cex=1.5)

points(CMalfruit.x~Group.3,data=compil_idn_sm_2011[compil_idn_sm_2011$Group.2=="A"&compil_idn_sm_2011$Group.1=="SM90",],type="l",lty=1,ljoin="round",lwd=1)

points(CMalfruit.x~Group.3,data=compil_idn_sm_2011[compil_idn_sm_2011$Group.2=="T"&compil_idn_sm_2011$Group.1=="SM90",],type="l",lty=2,lwd=1,ljoin="round")

# to draw the standard deviations

for (i in 1:5) {segments(compil_idn_sm_2011$Group.3[compil_idn_sm_2011$Group.2=="A"&compil_idn_sm_2011$Group.1=="SM90"][i],compil_idn_sm_2011$malatemmolKgMF.x[compil_idn_sm_2011$Group.2=="A"&compil_idn_sm_2011$Group.1=="SM90"][i]-compil_idn_sm_2011$malatemmolKgMF.y[compil_idn_sm_2011$Group.2=="A"&compil_idn_sm_2011$Group.1=="SM90"][i] ,compil_idn_sm_2011$Group.3[compil_idn_sm_2011$Group.2=="A"&compil_idn_sm_2011$Group.1=="SM90"][i],compil_idn_sm_2011$malatemmolKgMF.x[compil_idn_sm_2011$Group.2=="A"&compil_idn_sm_2011$Group.1=="SM90"][i]+compil_idn_sm_2011$malatemmolKgMF.y[compil_idn_sm_2011$Group.2=="A"&compil_idn_sm_2011$Group.1=="SM90"][i],lty=1)}

for (i in 1:5) {segments(compil_idn_sm_2011$Group.3[compil_idn_sm_2011$Group.2=="T"&compil_idn_sm_2011$Group.1=="SM90"][i],compil_idn_sm_2011$malatemmolKgMF.x[compil_idn_sm_2011$Group.2=="T"&compil_idn_sm_2011$Group.1=="SM90"][i]-compil_idn_sm_2011$malatemmolKgMF.y[compil_idn_sm_2011$Group.2=="T"&compil_idn_sm_2011$Group.1=="SM90"][i] ,compil_idn_sm_2011$Group.3[compil_idn_sm_2011$Group.2=="T"&compil_idn_sm_2011$Group.1=="SM90"][i],compil_idn_sm_2011$malatemmolKgMF.x[compil_idn_sm_2011$Group.2=="T"&compil_idn_sm_2011$Group.1=="SM90"][i]+compil_idn_sm_2011$malatemmolKgMF.y[compil_idn_sm_2011$Group.2=="T"&compil_idn_sm_2011$Group.1=="SM90"][i],lty=2)}

# JB

par(mar=c(0,0,0,0))

plot(malatemmolKgMF.x~Group.3,data=compil_jb_sm_2011[compil_jb_sm_2011$Group.2=="A"&compil_jb_sm_2011$Group.1=="SM90",],ylim=c(0,80),xlim=c(0,13),xlab="stade",ylab="malate (mmol/Kg FW)",cex.lab=1.3,font.lab=2,pch=16,las=1,tck=0.02,xaxt="n",cex=1.5,yaxt="n")

axis(side=1,tck=0.02,labels=FALSE)

axis(side=2,tck=0.02,labels=FALSE)

points(malatemmolKgMF.x~Group.3,data=compil_jb_sm_2011[compil_jb_sm_2011$Group.2=="T"&compil_jb_sm_2011$Group.1=="SM90",],pch=1,cex=1.5)

points(CMalfruit.x~Group.3,data=compil_jb_sm_2011[compil_jb_sm_2011$Group.2=="A"&compil_jb_sm_2011$Group.1=="SM90",],type="l",lty=1,ljoin="round",lwd=1)

points(CMalfruit.x~Group.3,data=compil_jb_sm_2011[compil_jb_sm_2011$Group.2=="T"&compil_jb_sm_2011$Group.1=="SM90",],type="l",lty=2,lwd=1,ljoin="round")

# to draw the standard deviations

for (i in 1:5) {segments(compil_jb_sm_2011$Group.3[compil_jb_sm_2011$Group.2=="A"&compil_jb_sm_2011$Group.1=="SM90"][i],compil_jb_sm_2011$malatemmolKgMF.x[compil_jb_sm_2011$Group.2=="A"&compil_jb_sm_2011$Group.1=="SM90"][i]-compil_jb_sm_2011$malatemmolKgMF.y[compil_jb_sm_2011$Group.2=="A"&compil_jb_sm_2011$Group.1=="SM90"][i] ,compil_jb_sm_2011$Group.3[compil_jb_sm_2011$Group.2=="A"&compil_jb_sm_2011$Group.1=="SM90"][i],compil_jb_sm_2011$malatemmolKgMF.x[compil_jb_sm_2011$Group.2=="A"&compil_jb_sm_2011$Group.1=="SM90"][i]+compil_jb_sm_2011$malatemmolKgMF.y[compil_jb_sm_2011$Group.2=="A"&compil_jb_sm_2011$Group.1=="SM90"][i],lty=1)}

for (i in 1:5) {segments(compil_jb_sm_2011$Group.3[compil_jb_sm_2011$Group.2=="T"&compil_jb_sm_2011$Group.1=="SM90"][i],compil_jb_sm_2011$malatemmolKgMF.x[compil_jb_sm_2011$Group.2=="T"&compil_jb_sm_2011$Group.1=="SM90"][i]-compil_jb_sm_2011$malatemmolKgMF.y[compil_jb_sm_2011$Group.2=="T"&compil_jb_sm_2011$Group.1=="SM90"][i] ,compil_jb_sm_2011$Group.3[compil_jb_sm_2011$Group.2=="T"&compil_jb_sm_2011$Group.1=="SM90"][i],compil_jb_sm_2011$malatemmolKgMF.x[compil_jb_sm_2011$Group.2=="T"&compil_jb_sm_2011$Group.1=="SM90"][i]+compil_jb_sm_2011$malatemmolKgMF.y[compil_jb_sm_2011$Group.2=="T"&compil_jb_sm_2011$Group.1=="SM90"][i],lty=2)}

# PL

par(mar=c(0,0,0,0))

plot(malatemmolKgMF.x~Group.3,data=compil_pl_sm_2011[compil_pl_sm_2011$Group.2=="A"&compil_pl_sm_2011$Group.1=="SM90",],ylim=c(0,80),xlim=c(0,13),xlab="stade",ylab="malate (mmol/Kg FW)",cex.lab=1.3,font.lab=2,pch=16,las=1,tck=0.02,xaxt="n",cex=1.5,yaxt="n")

axis(side=1,tck=0.02,labels=FALSE)

axis(side=2,tck=0.02,labels=FALSE)

points(malatemmolKgMF.x~Group.3,data=compil_pl_sm_2011[compil_pl_sm_2011$Group.2=="T"&compil_pl_sm_2011$Group.1=="SM90",],pch=1,cex=1.5)

points(CMalfruit.x~Group.3,data=compil_pl_sm_2011[compil_pl_sm_2011$Group.2=="A"&compil_pl_sm_2011$Group.1=="SM90",],type="l",lty=1,ljoin="round",lwd=1)

points(CMalfruit.x~Group.3,data=compil_pl_sm_2011[compil_pl_sm_2011$Group.2=="T"&compil_pl_sm_2011$Group.1=="SM90",],type="l",lty=2,lwd=1,ljoin="round")

# to draw the standard deviations

for (i in 1:5) {segments(compil_pl_sm_2011$Group.3[compil_pl_sm_2011$Group.2=="A"&compil_pl_sm_2011$Group.1=="SM90"][i],compil_pl_sm_2011$malatemmolKgMF.x[compil_pl_sm_2011$Group.2=="A"&compil_pl_sm_2011$Group.1=="SM90"][i]-compil_pl_sm_2011$malatemmolKgMF.y[compil_pl_sm_2011$Group.2=="A"&compil_pl_sm_2011$Group.1=="SM90"][i] ,compil_pl_sm_2011$Group.3[compil_pl_sm_2011$Group.2=="A"&compil_pl_sm_2011$Group.1=="SM90"][i],compil_pl_sm_2011$malatemmolKgMF.x[compil_pl_sm_2011$Group.2=="A"&compil_pl_sm_2011$Group.1=="SM90"][i]+compil_pl_sm_2011$malatemmolKgMF.y[compil_pl_sm_2011$Group.2=="A"&compil_pl_sm_2011$Group.1=="SM90"][i],lty=1)}

for (i in 1:5) {segments(compil_pl_sm_2011$Group.3[compil_pl_sm_2011$Group.2=="T"&compil_pl_sm_2011$Group.1=="SM90"][i],compil_pl_sm_2011$malatemmolKgMF.x[compil_pl_sm_2011$Group.2=="T"&compil_pl_sm_2011$Group.1=="SM90"][i]-compil_pl_sm_2011$malatemmolKgMF.y[compil_pl_sm_2011$Group.2=="T"&compil_pl_sm_2011$Group.1=="SM90"][i] ,compil_pl_sm_2011$Group.3[compil_pl_sm_2011$Group.2=="T"&compil_pl_sm_2011$Group.1=="SM90"][i],compil_pl_sm_2011$malatemmolKgMF.x[compil_pl_sm_2011$Group.2=="T"&compil_pl_sm_2011$Group.1=="SM90"][i]+compil_pl_sm_2011$malatemmolKgMF.y[compil_pl_sm_2011$Group.2=="T"&compil_pl_sm_2011$Group.1=="SM90"][i],lty=2)}

## 2012

# IDN

par(mar=c(0,0,0,0))

plot(malatemmolKgMF.x~Group.2,data=compil_idn_sm_ferti[compil_idn_sm_ferti$Group.1=="K+",],ylim=c(0,80),xlim=c(0,13),xlab="stade",ylab="malate (mmol/Kg FW)",cex.lab=1.3,font.lab=2,pch=17,las=1,tck=0.02,xaxt="n",cex=1.5,yaxt="n")

axis(side=1,tck=0.02,labels=FALSE)

axis(side=2,tck=0.02,labels=FALSE)

points(malatemmolKgMF.x~Group.2,data=compil_idn_sm_ferti[compil_idn_sm_ferti$Group.1=="K-",],pch=2,cex=1.5)

points(CMalfruit.x~Group.2,data=compil_idn_sm_ferti[compil_idn_sm_ferti$Group.1=="K+",],type="l",lty=1,ljoin="round",lwd=1)

points(CMalfruit.x~Group.2,data=compil_idn_sm_ferti[compil_idn_sm_ferti$Group.1=="K-",],type="l",lty=2,lwd=1,ljoin="round")

# to draw the standard deviations

for (i in 1:5) {segments(compil_idn_sm_ferti$Group.2[compil_idn_sm_ferti$Group.1=="K+"][i],compil_idn_sm_ferti$malatemmolKgMF.x[compil_idn_sm_ferti$Group.1=="K+"][i]-compil_idn_sm_ferti$malatemmolKgMF.y[compil_idn_sm_ferti$Group.1=="K+"][i] ,compil_idn_sm_ferti$Group.2[compil_idn_sm_ferti$Group.1=="K+"][i],compil_idn_sm_ferti$malatemmolKgMF.x[compil_idn_sm_ferti$Group.1=="K+"][i]+compil_idn_sm_ferti$malatemmolKgMF.y[compil_idn_sm_ferti$Group.1=="K+"][i],lty=1)}

for (i in 1:5) {segments(compil_idn_sm_ferti$Group.2[compil_idn_sm_ferti$Group.1=="K-"][i],compil_idn_sm_ferti$malatemmolKgMF.x[compil_idn_sm_ferti$Group.1=="K-"][i]-compil_idn_sm_ferti$malatemmolKgMF.y[compil_idn_sm_ferti$Group.1=="K-"][i] ,compil_idn_sm_ferti$Group.2[compil_idn_sm_ferti$Group.1=="K-"][i],compil_idn_sm_ferti$malatemmolKgMF.x[compil_idn_sm_ferti$Group.1=="K-"][i]+compil_idn_sm_ferti$malatemmolKgMF.y[compil_idn_sm_ferti$Group.1=="K-"][i],lty=2)}

points(1,80,pch=17,cex=1.5)

segments(0,80,2,80,lty=1)

text(2.5,80,adj=0,"HF",cex=1,font=2)

points(1,75,pch=2,cex=1.5)

segments(0,75,2,75,lty=2)

text(2.5,75,adj=0,"NF",cex=1,font=2)

# JB

par(mar=c(0,0,0,0))

plot(malatemmolKgMF.x~Group.2,data=compil_jb_sm_ferti[compil_idn_sm_ferti$Group.1=="K+",],ylim=c(0,80),xlim=c(0,13),xlab="stade",ylab="malate (mmol/Kg FW)",cex.lab=1.3,font.lab=2,pch=17,las=1,tck=0.02,xaxt="n",cex=1.5,yaxt="n")

axis(side=1,tck=0.02,labels=FALSE)

axis(side=2,tck=0.02,labels=FALSE)

points(malatemmolKgMF.x~Group.2,data=compil_jb_sm_ferti[compil_jb_sm_ferti$Group.1=="K-",],pch=2,cex=1.5)

points(CMalfruit.x~Group.2,data=compil_jb_sm_ferti[compil_jb_sm_ferti$Group.1=="K+",],type="l",lty=1,ljoin="round",lwd=1)

points(CMalfruit.x~Group.2,data=compil_jb_sm_ferti[compil_jb_sm_ferti$Group.1=="K-",],type="l",lty=2,lwd=1,ljoin="round")

# to draw the standard deviations

for (i in 1:5) {segments(compil_jb_sm_ferti$Group.2[compil_jb_sm_ferti$Group.1=="K+"][i],compil_jb_sm_ferti$malatemmolKgMF.x[compil_jb_sm_ferti$Group.1=="K+"][i]-compil_jb_sm_ferti$malatemmolKgMF.y[compil_jb_sm_ferti$Group.1=="K+"][i] ,compil_jb_sm_ferti$Group.2[compil_jb_sm_ferti$Group.1=="K+"][i],compil_jb_sm_ferti$malatemmolKgMF.x[compil_jb_sm_ferti$Group.1=="K+"][i]+compil_jb_sm_ferti$malatemmolKgMF.y[compil_jb_sm_ferti$Group.1=="K+"][i],lty=1)}

for (i in 1:5) {segments(compil_jb_sm_ferti$Group.2[compil_jb_sm_ferti$Group.1=="K-"][i],compil_jb_sm_ferti$malatemmolKgMF.x[compil_jb_sm_ferti$Group.1=="K-"][i]-compil_jb_sm_ferti$malatemmolKgMF.y[compil_jb_sm_ferti$Group.1=="K-"][i] ,compil_jb_sm_ferti$Group.2[compil_jb_sm_ferti$Group.1=="K-"][i],compil_jb_sm_ferti$malatemmolKgMF.x[compil_jb_sm_ferti$Group.1=="K-"][i]+compil_jb_sm_ferti$malatemmolKgMF.y[compil_jb_sm_ferti$Group.1=="K-"][i],lty=2)}

# PL

par(mar=c(0,0,0,0))

plot(malatemmolKgMF.x~Group.2,data=compil_pl_sm_ferti[compil_idn_sm_ferti$Group.1=="K+",],ylim=c(0,80),xlim=c(0,13),xlab="stade",ylab="malate (mmol/Kg FW)",cex.lab=1.3,font.lab=2,pch=17,las=1,tck=0.02,xaxt="n",cex=1.5,yaxt="n")

axis(side=1,tck=0.02,labels=FALSE)

axis(side=2,tck=0.02,labels=FALSE)

points(malatemmolKgMF.x~Group.2,data=compil_pl_sm_ferti[compil_pl_sm_ferti$Group.1=="K-",],pch=2,cex=1.5)

points(CMalfruit.x~Group.2,data=compil_pl_sm_ferti[compil_pl_sm_ferti$Group.1=="K+",],type="l",lty=1,ljoin="round",lwd=1)

points(CMalfruit.x~Group.2,data=compil_pl_sm_ferti[compil_pl_sm_ferti$Group.1=="K-",],type="l",lty=2,lwd=1,ljoin="round")

# to draw the standard deviations

for (i in 1:5) {segments(compil_pl_sm_ferti$Group.2[compil_pl_sm_ferti$Group.1=="K+"][i],compil_pl_sm_ferti$malatemmolKgMF.x[compil_pl_sm_ferti$Group.1=="K+"][i]-compil_pl_sm_ferti$malatemmolKgMF.y[compil_pl_sm_ferti$Group.1=="K+"][i] ,compil_pl_sm_ferti$Group.2[compil_pl_sm_ferti$Group.1=="K+"][i],compil_pl_sm_ferti$malatemmolKgMF.x[compil_pl_sm_ferti$Group.1=="K+"][i]+compil_pl_sm_ferti$malatemmolKgMF.y[compil_pl_sm_ferti$Group.1=="K+"][i],lty=1)}

for (i in 1:5) {segments(compil_pl_sm_ferti$Group.2[compil_pl_sm_ferti$Group.1=="K-"][i],compil_pl_sm_ferti$malatemmolKgMF.x[compil_pl_sm_ferti$Group.1=="K-"][i]-compil_pl_sm_ferti$malatemmolKgMF.y[compil_pl_sm_ferti$Group.1=="K-"][i] ,compil_pl_sm_ferti$Group.2[compil_pl_sm_ferti$Group.1=="K-"][i],compil_pl_sm_ferti$malatemmolKgMF.x[compil_pl_sm_ferti$Group.1=="K-"][i]+compil_pl_sm_ferti$malatemmolKgMF.y[compil_pl_sm_ferti$Group.1=="K-"][i],lty=2)}

**R SCRIPT TO MAKE THE GRAPHICS OF OBSERVATIONS AND PREDICTIONS IN 2011 AND 2012 FOR THE GROWTH STAGE**

## Suppression of aberrant predictions

Malsimidn_sc_2011<-Malsimidn_sc_2011[Malsimidn_sc_2011$DPSI>=0,]

Malsimjb_sc_2011<-Malsimjb_sc_2011[Malsimjb_sc_2011$DPSI>=0,]

Malsimpl_sc_2011<-Malsimpl_sc_2011[Malsimpl_sc_2011$DPSI>=0,]

Malsimidn_sc_ferti<-Malsimidn_sc_ferti[Malsimidn_sc_ferti$DPSI>=0,]

Malsimjb_sc_ferti<-Malsimjb_sc_ferti[Malsimjb_sc_ferti$DPSI>=0,]

Malsimpl_sc_ferti<-Malsimpl_sc_ferti[Malsimpl_sc_ferti$DPSI>=0,]

####### Calculation of the mean and sd of the predictions by genotype and by growth stage

### IDN 2011

moy_idn_sc_2011<-aggregate(Malsimidn_sc_2011[,20:34],by=list(Malsimidn_sc_2011$treatment,Malsimidn_sc_2011$sample),FUN=mean)

ect_idn_sc_2011<-aggregate(Malsimidn_sc_2011[,20:34],by=list(Malsimidn_sc_2011$treatment,Malsimidn_sc_2011$sample),FUN=sd)

compil_idn_sc_2011<-merge(moy_idn_sc_2011,ect_idn_sc_2011,by=c("Group.1","Group.2"))

### JB 2011

moy_jb_sc_2011<-aggregate(Malsimjb_sc_2011[,20:34],by=list(Malsimjb_sc_2011$treatment,Malsimjb_sc_2011$sample),FUN=mean)

ect_jb_sc_2011<-aggregate(Malsimjb_sc_2011[,20:34],by=list(Malsimjb_sc_2011$treatment,Malsimjb_sc_2011$sample),FUN=sd)

compil_jb_sc_2011<-merge(moy_jb_sc_2011,ect_jb_sc_2011,by=c("Group.1","Group.2"))

### PL 2011

moy_pl_sc_2011<-aggregate(Malsimpl_sc_2011[,20:34],by=list(Malsimpl_sc_2011$treatment,Malsimpl_sc_2011$sample),FUN=mean)

ect_pl_sc_2011<-aggregate(Malsimpl_sc_2011[,20:34],by=list(Malsimpl_sc_2011$treatment,Malsimpl_sc_2011$sample),FUN=sd)

compil_pl_sc_2011<-merge(moy_pl_sc_2011,ect_pl_sc_2011,by=c("Group.1","Group.2"))

### IDN 2012

moy_idn_sc_ferti<-aggregate(Malsimidn_sc_ferti[,20:34],by=list(Malsimidn_sc_ferti$Ferti,Malsimidn_sc_ferti$sample),FUN=mean)

ect_idn_sc_ferti<-aggregate(Malsimidn_sc_ferti[,20:34],by=list(Malsimidn_sc_ferti$Ferti,Malsimidn_sc_ferti$sample),FUN=sd)

compil_idn_sc_ferti<-merge(moy_idn_sc_ferti,ect_idn_sc_ferti,by=c("Group.1","Group.2"))

### JB 2012

moy_jb_sc_ferti<-aggregate(Malsimjb_sc_ferti[,20:34],by=list(Malsimjb_sc_ferti$Ferti,Malsimjb_sc_ferti$sample),FUN=mean)

ect_jb_sc_ferti<-aggregate(Malsimjb_sc_ferti[,20:34],by=list(Malsimjb_sc_ferti$Ferti,Malsimjb_sc_ferti$sample),FUN=sd)

compil_jb_sc_ferti<-merge(moy_jb_sc_ferti,ect_jb_sc_ferti,by=c("Group.1","Group.2"))

### PL 2012

moy_pl_sc_ferti<-aggregate(Malsimpl_sc_ferti[,20:34],by=list(Malsimpl_sc_ferti$Ferti,Malsimpl_sc_ferti$sample),FUN=mean)

ect_pl_sc_ferti<-aggregate(Malsimpl_sc_ferti[,20:34],by=list(Malsimpl_sc_ferti$Ferti,Malsimpl_sc_ferti$sample),FUN=sd)

compil_pl_sc_ferti<-merge(moy_pl_sc_ferti,ect_pl_sc_ferti,by=c("Group.1","Group.2"))

# to sort data by increasing growing stage (in order to have a nice line graphic)

compil_idn_sc_2011<-compil_idn_sc_2011[order(compil_idn_sc_2011$Group.1,compil_idn_sc_2011$Group.2),]

compil_jb_sc_2011<-compil_jb_sc_2011[order(compil_jb_sc_2011$Group.1,compil_jb_sc_2011$Group.2),]

compil_pl_sc_2011<-compil_pl_sc_2011[order(compil_pl_sc_2011$Group.1,compil_pl_sc_2011$Group.2),]

compil_idn_sc_ferti<-compil_idn_sc_ferti[order(compil_idn_sc_ferti$Group.1,compil_idn_sc_ferti$Group.2),]

compil_jb_sc_ferti<-compil_jb_sc_ferti[order(compil_jb_sc_ferti$Group.1,compil_jb_sc_ferti$Group.2),]

compil_pl_sc_ferti<-compil_pl_sc_ferti[order(compil_pl_sc_ferti$Group.1,compil_pl_sc_ferti$Group.2),]

###### Drawing the graphics

layout(matrix(1:6, 2, 3,byrow=TRUE))

layout.show(6)

## 2011

# IDN

par(mar=c(0,0,0,0))

plot(malatemmolKgMF.x~Group.2,data=compil_idn_sc_2011[compil_idn_sc_2011$Group.1=="T",],ylim=c(0,30),xlim=c(3,8),xlab="stade",ylab="malate (mmol/Kg FW)",cex.lab=1.3,font.lab=2,pch=1,las=1,tck=0.02,lab=c(6,7,0),xaxt="n",cex=1.5)

axis(side=1,tck=0.02,labels=FALSE)

points(malatemmolKgMF.x~Group.2,data=compil_idn_sc_2011[compil_idn_sc_2011$Group.1=="A",],pch=16,cex=1.5)

points(CMalfruit.x~Group.2,data=compil_idn_sc_2011[compil_idn_sc_2011$Group.1=="T",],type="l",lty=2,lwd=1,ljoin="round")

points(CMalfruit.x~Group.2,data=compil_idn_sc_2011[compil_idn_sc_2011$Group.1=="A",],type="l",lty=1,ljoin="round",lwd=1)

# to draw the standard deviations

for (i in 1:6) {segments(compil_idn_sc_2011$Group.2[compil_idn_sc_2011$Group.1=="T"][i],compil_idn_sc_2011$malatemmolKgMF.x[compil_idn_sc_2011$Group.1=="T"][i]-compil_idn_sc_2011$malatemmolKgMF.y[compil_idn_sc_2011$Group.1=="T"][i] ,compil_idn_sc_2011$Group.2[compil_idn_sc_2011$Group.1=="T"][i],compil_idn_sc_2011$malatemmolKgMF.x[compil_idn_sc_2011$Group.1=="T"][i]+compil_idn_sc_2011$malatemmolKgMF.y[compil_idn_sc_2011$Group.1=="T"][i],lty=2)}

for (i in 1:6) {segments(compil_idn_sc_2011$Group.2[compil_idn_sc_2011$Group.1=="A"][i],compil_idn_sc_2011$malatemmolKgMF.x[compil_idn_sc_2011$Group.1=="A"][i]-compil_idn_sc_2011$malatemmolKgMF.y[compil_idn_sc_2011$Group.1=="A"][i] ,compil_idn_sc_2011$Group.2[compil_idn_sc_2011$Group.1=="A"][i],compil_idn_sc_2011$malatemmolKgMF.x[compil_idn_sc_2011$Group.1=="A"][i]+compil_idn_sc_2011$malatemmolKgMF.y[compil_idn_sc_2011$Group.1=="A"][i],lty=1)}

# to draw the legend

points(3.5,30,pch=16,cex=1.5)

segments(3,30,4,30,lty=1)

text(4.3,30,adj=0,"LL",cex=1,font=2)

points(3.5,28,pch=1,bg="grey",cex=1.5)

segments(3,28,4,28,lty=2)

text(4.3,28,adj=0,"HL",cex=1,font=2)

# JB

par(mar=c(0,0,0,0))

plot(malatemmolKgMF.x~Group.2,data=compil_jb_sc_2011[compil_jb_sc_2011$Group.1=="T",],ylim=c(0,30),xlim=c(3,6),xlab="stade",ylab="malate (mmol/Kg FW)",cex.lab=1.3,font.lab=2,pch=1,las=1,tck=0.02,lab=c(4,7,0),xaxt="n",cex=1.5,yaxt="n")

axis(side=1,tck=0.02,labels=FALSE)

axis(side=2,tck=0.02,labels=FALSE)

points(malatemmolKgMF.x~Group.2,data=compil_jb_sc_2011[compil_jb_sc_2011$Group.1=="A",],pch=16,cex=1.5)

points(CMalfruit.x~Group.2,data=compil_jb_sc_2011[compil_jb_sc_2011$Group.1=="T",],type="l",lty=2,lwd=1,ljoin="round")

points(CMalfruit.x~Group.2,data=compil_jb_sc_2011[compil_jb_sc_2011$Group.1=="A",],type="l",lty=1,ljoin="round",lwd=1)

# to draw the standard deviations

for (i in 1:4) {segments(compil_jb_sc_2011$Group.2[compil_jb_sc_2011$Group.1=="T"][i],compil_jb_sc_2011$malatemmolKgMF.x[compil_jb_sc_2011$Group.1=="T"][i]-compil_jb_sc_2011$malatemmolKgMF.y[compil_jb_sc_2011$Group.1=="T"][i] ,compil_jb_sc_2011$Group.2[compil_jb_sc_2011$Group.1=="T"][i],compil_jb_sc_2011$malatemmolKgMF.x[compil_jb_sc_2011$Group.1=="T"][i]+compil_jb_sc_2011$malatemmolKgMF.y[compil_jb_sc_2011$Group.1=="T"][i],lty=2)}

for (i in 1:4) {segments(compil_jb_sc_2011$Group.2[compil_jb_sc_2011$Group.1=="A"][i],compil_jb_sc_2011$malatemmolKgMF.x[compil_jb_sc_2011$Group.1=="A"][i]-compil_jb_sc_2011$malatemmolKgMF.y[compil_jb_sc_2011$Group.1=="A"][i] ,compil_jb_sc_2011$Group.2[compil_jb_sc_2011$Group.1=="A"][i],compil_jb_sc_2011$malatemmolKgMF.x[compil_jb_sc_2011$Group.1=="A"][i]+compil_jb_sc_2011$malatemmolKgMF.y[compil_jb_sc_2011$Group.1=="A"][i],lty=1)}

# PL

par(mar=c(0,0,0,0))

plot(malatemmolKgMF.x~Group.2,data=compil_pl_sc_2011[compil_pl_sc_2011$Group.1=="T",],ylim=c(0,30),xlim=c(3,7),xlab="stade",ylab="malate (mmol/Kg FW)",cex.lab=1.3,font.lab=2,pch=1,las=1,tck=0.02,lab=c(5,7,0),xaxt="n",cex=1.5,yaxt="n")

axis(side=1,tck=0.02,labels=FALSE)

axis(side=2,tck=0.02,labels=FALSE)

points(malatemmolKgMF.x~Group.2,data=compil_pl_sc_2011[compil_pl_sc_2011$Group.1=="A",],pch=16,cex=1.5)

points(CMalfruit.x~Group.2,data=compil_pl_sc_2011[compil_pl_sc_2011$Group.1=="T",],type="l",lty=2,lwd=1,ljoin="round")

points(CMalfruit.x~Group.2,data=compil_pl_sc_2011[compil_pl_sc_2011$Group.1=="A",],type="l",lty=1,ljoin="round",lwd=1)

# to draw the standard deviations

for (i in 1:5) {segments(compil_pl_sc_2011$Group.2[compil_pl_sc_2011$Group.1=="T"][i],compil_pl_sc_2011$malatemmolKgMF.x[compil_pl_sc_2011$Group.1=="T"][i]-compil_pl_sc_2011$malatemmolKgMF.y[compil_pl_sc_2011$Group.1=="T"][i] ,compil_pl_sc_2011$Group.2[compil_pl_sc_2011$Group.1=="T"][i],compil_pl_sc_2011$malatemmolKgMF.x[compil_pl_sc_2011$Group.1=="T"][i]+compil_pl_sc_2011$malatemmolKgMF.y[compil_pl_sc_2011$Group.1=="T"][i],lty=2)}

for (i in 1:5) {segments(compil_pl_sc_2011$Group.2[compil_pl_sc_2011$Group.1=="A"][i],compil_pl_sc_2011$malatemmolKgMF.x[compil_pl_sc_2011$Group.1=="A"][i]-compil_pl_sc_2011$malatemmolKgMF.y[compil_pl_sc_2011$Group.1=="A"][i] ,compil_pl_sc_2011$Group.2[compil_pl_sc_2011$Group.1=="A"][i],compil_pl_sc_2011$malatemmolKgMF.x[compil_pl_sc_2011$Group.1=="A"][i]+compil_pl_sc_2011$malatemmolKgMF.y[compil_pl_sc_2011$Group.1=="A"][i],lty=1)}

## 2012

# IDN

par(mar=c(0,0,0,0))

plot(malatemmolKgMF.x~Group.2,data=compil_idn_sc_ferti[compil_idn_sc_ferti$Group.1=="K+",],ylim=c(0,30),xlim=c(3,8),xlab="stade",ylab="malate (mmol/Kg FW)",cex.lab=1.3,font.lab=2,pch=17,las=1,tck=0.02,lab=c(6,7,0),xaxt="n",cex=1.5)

axis(side=1,tck=0.02,labels=FALSE)

points(malatemmolKgMF.x~Group.2,data=compil_idn_sc_ferti[compil_idn_sc_ferti$Group.1=="K-",],pch=2,cex=1.5)

points(CMalfruit.x~Group.2,data=compil_idn_sc_ferti[compil_idn_sc_ferti$Group.1=="K+",],type="l",lty=1,ljoin="round",lwd=1)

points(CMalfruit.x~Group.2,data=compil_idn_sc_ferti[compil_idn_sc_ferti$Group.1=="K-",],type="l",lty=2,lwd=1,ljoin="round")

# to draw the standard deviations

for (i in 1:6) {segments(compil_idn_sc_ferti$Group.2[compil_idn_sc_ferti$Group.1=="K+"][i],compil_idn_sc_ferti$malatemmolKgMF.x[compil_idn_sc_ferti$Group.1=="K+"][i]-compil_idn_sc_ferti$malatemmolKgMF.y[compil_idn_sc_ferti$Group.1=="K+"][i] ,compil_idn_sc_ferti$Group.2[compil_idn_sc_ferti$Group.1=="K+"][i],compil_idn_sc_ferti$malatemmolKgMF.x[compil_idn_sc_ferti$Group.1=="K+"][i]+compil_idn_sc_ferti$malatemmolKgMF.y[compil_idn_sc_ferti$Group.1=="K+"][i],lty=1)}

for (i in 1:6) {segments(compil_idn_sc_ferti$Group.2[compil_idn_sc_ferti$Group.1=="K-"][i],compil_idn_sc_ferti$malatemmolKgMF.x[compil_idn_sc_ferti$Group.1=="K-"][i]-compil_idn_sc_ferti$malatemmolKgMF.y[compil_idn_sc_ferti$Group.1=="K-"][i] ,compil_idn_sc_ferti$Group.2[compil_idn_sc_ferti$Group.1=="K-"][i],compil_idn_sc_ferti$malatemmolKgMF.x[compil_idn_sc_ferti$Group.1=="K-"][i]+compil_idn_sc_ferti$malatemmolKgMF.y[compil_idn_sc_ferti$Group.1=="K-"][i],lty=2)}

# to draw the legend

points(3.5,30,pch=17,cex=1.5)

segments(3,30,4,30,lty=1)

text(4.3,30,adj=0,"HF",cex=1,font=2)

points(3.5,28,pch=2,cex=1.5)

segments(3,28,4,28,lty=2)

text(4.3,28,adj=0,"NF",cex=1,font=2)

# JB

par(mar=c(0,0,0,0))

plot(malatemmolKgMF.x~Group.2,data=compil_jb_sc_ferti[compil_jb_sc_ferti$Group.1=="K+",],ylim=c(0,30),xlim=c(3,6),xlab="stade",ylab="malate (mmol/Kg FW)",cex.lab=1.3,font.lab=2,pch=17,las=1,tck=0.02,lab=c(4,7,0),xaxt="n",cex=1.5,yaxt="n")

axis(side=1,tck=0.02,labels=FALSE)

axis(side=2,tck=0.02,labels=FALSE)

points(malatemmolKgMF.x~Group.2,data=compil_jb_sc_ferti[compil_jb_sc_ferti$Group.1=="K-",],pch=2,cex=1.5)

points(CMalfruit.x~Group.2,data=compil_jb_sc_ferti[compil_jb_sc_ferti$Group.1=="K+",],type="l",lty=1,ljoin="round",lwd=1)

points(CMalfruit.x~Group.2,data=compil_jb_sc_ferti[compil_jb_sc_ferti$Group.1=="K-",],type="l",lty=2,lwd=1,ljoin="round")

# to draw the standard deviations

for (i in 1:4) {segments(compil_jb_sc_ferti$Group.2[compil_jb_sc_ferti$Group.1=="K+"][i],compil_jb_sc_ferti$malatemmolKgMF.x[compil_jb_sc_ferti$Group.1=="K+"][i]-compil_jb_sc_ferti$malatemmolKgMF.y[compil_jb_sc_ferti$Group.1=="K+"][i] ,compil_jb_sc_ferti$Group.2[compil_jb_sc_ferti$Group.1=="K+"][i],compil_jb_sc_ferti$malatemmolKgMF.x[compil_jb_sc_ferti$Group.1=="K+"][i]+compil_jb_sc_ferti$malatemmolKgMF.y[compil_jb_sc_ferti$Group.1=="K+"][i],lty=1)}

for (i in 1:4) {segments(compil_jb_sc_ferti$Group.2[compil_jb_sc_ferti$Group.1=="K-"][i],compil_jb_sc_ferti$malatemmolKgMF.x[compil_jb_sc_ferti$Group.1=="K-"][i]-compil_jb_sc_ferti$malatemmolKgMF.y[compil_jb_sc_ferti$Group.1=="K-"][i] ,compil_jb_sc_ferti$Group.2[compil_jb_sc_ferti$Group.1=="K-"][i],compil_jb_sc_ferti$malatemmolKgMF.x[compil_jb_sc_ferti$Group.1=="K-"][i]+compil_jb_sc_ferti$malatemmolKgMF.y[compil_jb_sc_ferti$Group.1=="K-"][i],lty=2)}

# PL

par(mar=c(0,0,0,0))

plot(malatemmolKgMF.x~Group.2,data=compil_pl_sc_ferti[compil_pl_sc_ferti$Group.1=="K+",],ylim=c(0,30),xlim=c(3,7),xlab="stade",ylab="malate (mmol/Kg FW)",cex.lab=1.3,font.lab=2,pch=17,las=1,tck=0.02,lab=c(5,7,0),xaxt="n",cex=1.5,yaxt="n")

axis(side=1,tck=0.02,labels=FALSE)

axis(side=2,tck=0.02,labels=FALSE)

points(malatemmolKgMF.x~Group.2,data=compil_pl_sc_ferti[compil_pl_sc_ferti$Group.1=="K-",],pch=2,cex=1.5)

points(CMalfruit.x~Group.2,data=compil_pl_sc_ferti[compil_pl_sc_ferti$Group.1=="K+",],type="l",lty=1,ljoin="round",lwd=1)

points(CMalfruit.x~Group.2,data=compil_pl_sc_ferti[compil_pl_sc_ferti$Group.1=="K-",],type="l",lty=2,lwd=1,ljoin="round")

# to draw the standard deviations

for (i in 1:4) {segments(compil_pl_sc_ferti$Group.2[compil_pl_sc_ferti$Group.1=="K+"][i],compil_pl_sc_ferti$malatemmolKgMF.x[compil_pl_sc_ferti$Group.1=="K+"][i]-compil_pl_sc_ferti$malatemmolKgMF.y[compil_pl_sc_ferti$Group.1=="K+"][i] ,compil_pl_sc_ferti$Group.2[compil_pl_sc_ferti$Group.1=="K+"][i],compil_pl_sc_ferti$malatemmolKgMF.x[compil_pl_sc_ferti$Group.1=="K+"][i]+compil_pl_sc_ferti$malatemmolKgMF.y[compil_pl_sc_ferti$Group.1=="K+"][i],lty=1)}

for (i in 1:4) {segments(compil_pl_sc_ferti$Group.2[compil_pl_sc_ferti$Group.1=="K-"][i],compil_pl_sc_ferti$malatemmolKgMF.x[compil_pl_sc_ferti$Group.1=="K-"][i]-compil_pl_sc_ferti$malatemmolKgMF.y[compil_pl_sc_ferti$Group.1=="K-"][i] ,compil_pl_sc_ferti$Group.2[compil_pl_sc_ferti$Group.1=="K-"][i],compil_pl_sc_ferti$malatemmolKgMF.x[compil_pl_sc_ferti$Group.1=="K-"][i]+compil_pl_sc_ferti$malatemmolKgMF.y[compil_pl_sc_ferti$Group.1=="K-"][i],lty=2)}
